# Synthesis, DNA binding and biological evaluation of benzimidazole Schiff base ligands and their metal(ii) complexes†

**DOI:** 10.1039/d3ra00982c

**Published:** 2023-04-17

**Authors:** Khalid Mahmood, Zareen Akhter, Fouzia Perveen, Aisha, Muneeba Bibi, Hammad Ismail, Nida Tabassum, Sammer Yousuf, Ahmad Raza Ashraf, Muhammad Abdul Qayyum

**Affiliations:** a Department of Chemistry, Quaid-i-Azam University Islamabad Pakistan khalid42_edu@yahoo.com zareenakhter@yahoo.com; b Research Centre for Modeling and Simulations, National University of Sciences and Technology (NUST) Islamabad Pakistan; c Department of Biochemistry, Quaid-i-Azam University Islamabad Pakistan; d Department of Biochemistry and Biotechnology, University of Gujrat Gujrat Pakistan; e H.E.J. Research Institute of Chemistry, International Center for Chemical and Biological Sciences, University of Karachi Karachi Pakistan; f Department of Chemistry, Division of Science and Technology, University of Education Lahore Pakistan

## Abstract

Two novel benzimidazole ligands (*E*)-2-((4-(1*H*-benzo[*d*]imidazole-2-yl)phenylimino)methyl)-6-bromo-4-chlorophenol (L_1_) and (*E*)-1-((4-(1*H*-benzo[*d*]imidazole-2-yl)phenylimino)methyl)naphthalene-2-ol (L_2_) with their corresponding Cu(ii), Ni(ii), Pd(ii) and Zn(ii) complexes were designed and synthesized. The compounds were characterized by elemental, IR, and NMR (^1^H & ^13^C) spectral analyses. Molecular masses were determined by ESI-mass spectrometry, and the structure of ligand L_1_ was confirmed by single crystal X-ray diffraction analysis. Molecular docking was carried out for the theoretical investigation of DNA binding interactions. The results obtained were verified experimentally by UV/Visible absorption spectroscopy in conjunction with DNA thermal denaturation studies. It was observed that ligands (L_1_ and L_2_) and complexes (1–8) were moderate to strong DNA binders, as evident from the binding constants (*K*_b_). The value was found to be highest for complex 2 (3.27 × 10^5^ M^−1^) and lowest for 5 (6.40 × 10^3^ M^−1^). A cell line study revealed that breast cancer cells were less viable to the synthesized compounds compared to that of standard drugs, cisplatin and doxorubicin, at the same concentration. The compounds were also screened for *in vitro* antibacterial activity for which complex 2 showed a promising broad-spectrum effect against all tested strains of bacteria, almost in the proximity of the reference drug kanamycin, while the rest of the compounds displayed activity against selected strains.

## Abstract 2

Two novel benzimidazole ligands (*E*)-2-((4-(1*H*-benzo[*d*]imidazole-2-yl)phenylimino)methyl)-6-bromo-4-chlorophenol and (*E*)-1-((4-(1*H*-benzo[*d*]imidazole-2-yl)phenylimino)methyl) naphthalene-2-ol and their Cu(ii), Ni(ii), Pd(ii) and Zn(ii) complexes were designed and synthesized.

## Graphical abstract

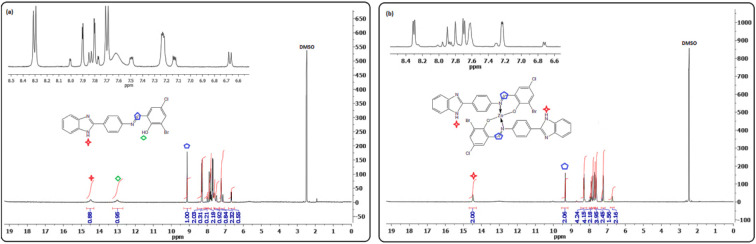

## 1. Introduction

Metal complexes are inherently endowed with extensive biological properties. Despite their scant implementation in the pharmaceutical industry as drugs, these compounds are widely used to unveil the structural behavior and functions of nucleic acids and promoters in various fascinating processes.^1^ Since the discovery of cisplatin in 1965 by serendipity,^2,3^ and its outstanding anticancer properties,^4^ a research interest has been evoked in the development of new metallo–organic complexes as chemotherapeutic agents. The most investigated metal complexes to date entail platinum and ruthenium central metal ions.^5,6^ However, owing to the high cost and adverse side effects associated with the aforementioned metal centers, first-row transition metal complexes are considered promising candidates for the design of potential anticancer agents.^7–9^ Definite coordination geometries and distinctive photophysical properties enhance the functionality of these binding agents.^10^ Most inorganic medicines implemented as antifungal, antibacterial, and antineoplastic drugs incorporate these metal ions.^11–13^

Along with metal, ligands also play a pivotal role in defining potential activity, which ranges from the recognition of a target site to the activity of any free ligand.^7^ Thus, the organic sphere around the metal core dictates binding with the target biopolymer, *i.e.* nucleic acid. Owing to the significance of organic moieties in tuning the propensity of metal complexes to bind with DNA, the oxygen and nitrogen donor benzimidazole Schiff bases seemed to be the strong candidates as chelating agents according to the literature.^14–16^ These are planar heterocyclic ligands with a structural scaffold similar to that found in purine bases of nucleic acid,^17,18^ which have an effective DNA binding ability. It has also been reported that the azomethine functionality of Schiff bases is responsible for various biological activities, such as antitumor, antimicrobial, and herbicidal activities.^19^ Additionally, the complexes of benzimidazole Schiff base ligands with metal ions are efficient DNA binders and exhibit stronger binding affinity than those of free ligands.^20–22^

There are three primary ways in which anticancer compounds interact with DNA: (i) electrostatic interaction, (ii) intercalation between base pairs, and (iii) minor and major DNA groove binding interaction.^6^

During intercalation, the compounds stack between adjacent DNA base pairs, leading to significant π-electron overlap. The forces that sustain the stability of the DNA-intercalator complex, even more than DNA alone, are van der Waals, hydrogen bonding, hydrophobic, and/or charge transfer forces.^21–25^ DNA intercalators are used in chemotherapeutic treatment to inhibit DNA replication in rapidly growing cancer cells. The DNA-intercalator complex is stabilized by π–π stacking interaction and is thus less sensitive to ionic strength relative to the two other binding modes (groove and external binding). Structural changes are induced in DNA by intercalators. Intercalation stabilizes, lengthens, stiffens, and unwinds the DNA double helix.^26^ For an intercalator to fit between base pairs, the DNA must dynamically open a space between its base pairs by unwinding. The degree of unwinding varies depending on the intercalator. The ethidium cation unwinds DNA by about 26° and proflavine by about 17°. These structural modifications can lead to functional changes, often leading to the inhibition of transcription and replication and DNA repair processes, which make intercalators potent mutagens.^27^

Intercalation is usually independent of the DNA sequence context (a slight GC specificity has been observed). This mode of binding is usually favored by the presence of an extended fused aromatic ligand, such as PHEHAT or DPPZ. With less extended aromatic systems, intercalation is usually prevented through the clashing of ancillary ligands with the phosphodiester backbone so that only partial intercalation can occur.

Owing to the role of organic ligands and chelates in DNA binding interactions, we planned to synthesize ligands and metal complexes (intercalators) with benzimidazole and azomethine functionalities along with the varying number of aromatic rings in their structure. A comparative DNA interaction study of organic intercalators was carried out using UV-vis absorption spectroscopy and a thermal melting study of DNA. It is well known that the DNA binding ability of complexes can initiate cytotoxicity in cancerous cells.^23,28^ Therefore, it is important to study the effect of synthesized complexes on cancerous cells. In this regard, the breast cancer cell line was used, and anticancer activities were investigated. Furthermore, the antimicrobial and preliminary cytotoxic studies of these compounds in normal shrimp cells made them an intriguing dimension of the research presented in this study.

## 2. Results and discussion

The heterocyclic benzimidazole Schiff base ligands (L_1_ and L_2_) and their corresponding metal complexes (1–8) were successfully synthesized. The compounds were structurally characterized using important spectroscopic techniques, such as FTIR, NMR (^1^H and ^13^C), high-resolution mass spectrometry, and elemental and single crystal X-ray diffraction analysis, which indicated the high purity of compounds. The analytical and physical data of the compounds are presented in Table 1. Moreover, the compounds were assessed for biological activity by performing various biological assays (antibacterial, antifungal, antitumor, and cytotoxic) to check their potential as safe antibiotic drugs. DNA binding studies of the compounds were carried out by absorption titrations and thermal melting studies of DNA.

**Table 1:** Analytical and physical data of benzimidazole Schiff base ligands and their metal complexes

Compound	Formula	Molecular weight	Color	Yield (%)	Melting point (°C)
A_1_	C_13_H_12_N_3_	210	Light pink	62	159
L_1_	C_20_H_14_N_3_OClBr	426	Orange	86	174
L_2_	C_24_H_18_N_3_O	364	Red	78	169
1	C_40_H_26_N_6_O_2_CuCl_2_Br_2_	914	Brown	83	>300
2	C_40_H_26_N_6_O_2_NiCl_2_Br_2_	910	Red	67	>300
3	C_40_H_26_N_6_O_2_PdCl_2_Br_2_	957	Brown	62	>300
4	C_40_H_26_N_6_O_2_ZnCl_2_Br_2_	916	Yellow	77	>300
5	C_48_H_34_N_6_O_2_Cu	789	Green	75	225
6	C_48_H_33_N_6_O_2_Ni	785	Dark red	68	>300
7	C_48_H_33_N_6_O_2_Pd	831	Brown	61	>300
8	C_48_H_33_N_6_O_2_Zn	791	Yellow	79	211

### 2.1 Infrared (IR) spectroscopy

The IR spectral data of precursor A_1_ (Table 2) revealed two absorption bands at 3436 and 3357 cm^−1^, which are attributed to the amino (NH_2_) group (Fig. S1†). The disappearance of these bands and the appearance of new strong bands at 1619 and 1613 cm^−1^ (for azomethine groups) in the spectrum of ligands L_1_ and L_2_, respectively, confirmed the successful conversion of amine functionality into an Schiff base moiety (Fig. S4a and c†). These absorption bands were shifted to the lower region (1619 to 1587 cm^−1^) in the spectrum of the corresponding metal complexes, which confirmed the coordination of the azomethine group through the nitrogen atom (N→M).^24^ This was further confirmed by the appearance of new bands in the region of 433–490 cm^−1^ due to *ν*(M–N) bond vibrations. Furthermore, the absence of broad bands at 3295 and 3203 cm^−1^ (owing to the OH moiety in the ligand structure) in the spectra of the complexes indicated the involvement of phenolic oxygen in bonding with a metal center. Further evidence regarding the binding of oxygen with metal ions was provided by the presence of bands around 540–576 cm^−1^ in the spectra of complexes^25^ (Fig. S4b, d, S2a–c, and S3a–c†).

**Table 2:** Characteristic IR stretching bands of benzimidazole Schiff base ligands and their metal complexes in cm^−1^

Compound	N–H (ring)	*ν* NH_2_ amine	*ν* OH	*ν* C=N (ring)	*ν* (C=N) azomethine	*ν* C–N (ring)	*ν* M–O	*ν* M–N
A_1_		3436, 3357	—	1623	—	1439	—	—
L_1_	3625	—	3295	—	1619	1434	—	—
L_2_	3618	—	3203	—	1613	1432	—	—
1	3620	—	—	—	1587	1447	569	490
2	3650	—	—	—	1587	1433	562	476
3	3656	—	—	—	1598	1435	576	461
4	3615	—	—	—	1594	1429	576	426
5	3622	—	—	—	1594	1435	540	433
6	3617	—	—	—	1587	1431	562	454
7	3615	—	—	—	1589	1429	554	433
8	3621	—	—	—	1594	1436	576	475

### 2.2 ^1^H and ^13^C NMR spectroscopy

**Fig. 1:**
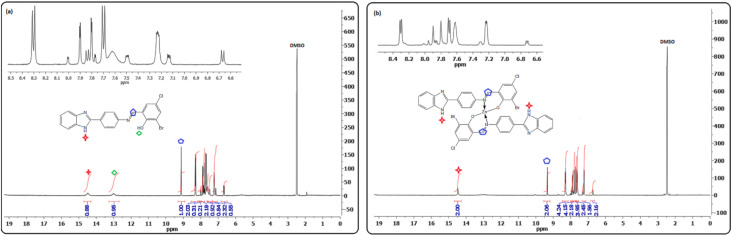
Representative ^1^H NMR spectrum of ligand L_1_ (a) and Zn(ii) complex 4 (b) in DMSO-d_6_.

Purity and structure confirmation were further validated by NMR spectroscopy. ^1^H NMR spectrum showed characteristic signals in the form of one proton singlet resonating at *δ* 9.13 and *δ* 9.73, attributable to the azomethine protons (–N=CH–) of L_1_ and L_2_, respectively. For the same ligands, the phenolic (–OH) and imine protons (–NH) were expectedly observed as two singlets at *δ*13.02 (L_1_) and *δ* 12.62 (L_2_) (–OH) as well as *δ* 14.49 (L_1_) and *δ* 15.74 (L_2_) (–NH) (Fig. 1a and S9a†).

The formation of Schiff base metal complexes was supported by observing a downfield shift of the azomethine proton from *δ* 9.11 (L_1_) and *δ* 9.73 (L_2_) to 9.52 (2), 9.50 (3), 9.46 (4), 9.76 (6), 10.03 (7), and 9.86 (8), as illustrated in Table 3. Strong evidence in favor of deprotonation and coordination of phenolic oxygen of ligands to metal ions was inferred from the disappearance of –OH proton singlets in the spectrum of complexes.^26,27^ Additionally, the number of protons obtained from the integrated spectral curves agreed well with those calculated from the CHN analyses.

**Table 3:** ^1^H NMR ^13^C NMR shifts of benzimidazole Schiff base ligands and their metal complexes in ppm

L_1_	C_20_H_14_N_3_OClBr	^1^H NMR (400 MHz, DMSO) *δ* 14.49 (s, 1H), 13.02 (s, 1H), 9.13 (s, 1H), 8.35 (d, *J* = 8.5 Hz, 2H), 8.01 (d, *J* = 2.5 Hz, 1H), 7.95–7.78 (m, 2H), 7.62 (s, 1H), 7.49 (d, *J* = 3.2 Hz, 1H), 7.18 (ddd, *J* = 38.6, 5.8, 3.0 Hz, 2H), 6.67 (d, *J* = 8.6 Hz, 1H). ^13^C NMR (101 MHz, DMSO) *δ* 165.12, 158.81, 150.13, 142.31–140.17, 134.83, 129.02, 122.13, 119.79, 113.42, 110.82
L_2_	C_24_H_18_N_3_O	^1^H NMR (300 MHz, DMSO) *δ* 15.74 (s, 1H), 12.62 (s, 1H) 9.73 (s, 1H), 8.55 (d, *J* = 8.4 Hz, 1H), 8.28 (d, *J* = 8.4, 2H), 8.95 (d, *J* = 9, 1H), 7.85 (m, 3H), 7.42 (d, *J* = 10.5 Hz, 2H), 7.36 (d, m, 2H), 7.02 (d, *J* = 9 Hz, 2H). ^13^C NMR (101 MHz, DMSO) *δ* 171.98, 162.59, 155.67, 152.56, 145.34, 137.90 133.65, 129.54, 128.69, 128.18, 128.09, 127.16, 124.14, 121.43, 120.96, 110.59, 109.18
1	C_40_H_26_N_6_O_2_CuCl_2_Br_2_	No data for copper complexes
2	C_40_H_26_N_6_O_2_NiCl_2_Br_2_	^1^H NMR (400 MHz, DMSO) *δ* 14.52 (s, 1H), 9.52 (s, 1H), 8.24 (dd, *J* = 24.3, 6.1 Hz, 4H), 7.83 (t, *J* = 22.3 Hz, 2H), 7.65 (dd, *J* = 27.7, 18.9 Hz, 2H), 7.35 (s, 1H), 7.26 (s, 1H), 7.06 (s, 2H), 6.94 (d, *J* = 7.7 Hz, 1H), 6.65 (d, *J* = 8.6 Hz, 1H). ^13^C NMR (101 MHz, DMSO) *δ* 170.51, 159.91, 151.65, 148.61–146.26, 142.19, 138.96, 137.03, 121.91–110.52
3	C_40_H_26_N_6_O_2_PdCl_2_Br_2_	^1^H NMR (400 MHz, DMSO) *δ* 14.41 (s, 1H), 9.50 (s, 1H), 7.91 (d, *J* = 7.2 Hz, 4H), 7.73–7.65 (m, 6H), 7.51 (s, 2H), 7.05 (d, *J* = 7.1 Hz, 4H), 6.82 (d, *J* = 8.2, 4H). ^13^C NMR (101 MHz, DMSO) *δ* 166.15, 158.79, 151.75, 148.71, 147.19, 145.82, 144.36, 135.03, 128.61, 127.52, 123.58–11.15
4	C_40_H_26_N_6_O_2_ZnCl_2_Br_2_	^1^H NMR (400 MHz, DMSO) *δ* 14.47 (s, 2H), 9.46 (s, 2H), 8.30 (d, *J* = 7.2 Hz, 4H), 8.01–7.87 (m, 4H), 7.81 (s, 2H), 7.70 (d, *J* = 7.1 Hz, 4H), 7.62 (s, 2H), 7.23 (dd, *J* = 17.2, 14.5 Hz, 2H), 6.74 (d, *J* = 7.1 Hz, 2H). ^13^C NMR (101 MHz, DMSO) *δ* 169.21, 158.92, 151.42, 149.73, 146.11, 144.23, 138.82, 137.51, 135.02, 129.41, 128.12, 123.20–111.07
5	C_48_H_34_N_6_O_2_Cu	No data for copper complexes
6	C_48_H_33_N_6_O_2_Ni	^1^H NMR (400 MHz, DMSO) *δ* 15.78 (s, 2H), 9.76 (s, 2H), 8.54 (s, 2H), 8.35–8.17 (m, 4H), 8.12 (d, *J* = 7.1 Hz, 2H), 7.95 (s, 2H), 7.83 (d, *J* = 22.4 Hz, 2H), 7.69 (d, *J* = 6.7 Hz, 4H), 7.63–7.49 (m, 6H), 7.38–7.10 (m, 4H), 7.10–6.83 (m, 2H). ^13^C NMR (101 MHz, CDCl_3_) *δ* 181.68, 164.21, 154.69, 150.07, 146.33, 144.0, 138.16, 132.30, 130.50, 129.50, 125.06, 124.03, 123.54–123.41, 122.03, 113.64–113.45, 110.02, 108.39
7	C_48_H_33_N_6_O_2_Pd	^1^H NMR (400 MHz, DMSO) *δ* 15.91 (s, 2H), 10.03 (s, 2H), 8.58 (d, *J* = 8.8 Hz, 2H), 8.31 (s, 4H), 8.12 (m, 2H), 7.95 (dd, *J* = 34.4, 25.3 Hz, 2H), 7.84 (d, *J* = 7.4 Hz, 4H), 7.65 (d, *J* = 36.5 Hz, 6H), 7.52 (m, 2H), 7.40 (m, 2H), 7.07 (d, *J* = 9.1 Hz, 4H). ^13^C NMR (101 MHz, DMSO) *δ* 182.05, 169.15, 155.54, 150.72, 145.03, 137.90, 133.65, 129.54, 128.69–128.08, 127.14, 124.28–124.13, 122.91, 121.37, 109.14
8	C_48_H_33_N_6_O_2_Zn	^1^H NMR (400 MHz, DMSO) *δ* 15.75 (s, 2H), 9.86 (s, 2H), 8.56 (d, *J* = 8.8 Hz, 2H), 8.32 (s, 4H), 8.13–8.10 (m, 2H), 8.05–7.95 (m, 2H), 7.84 (d, *J* = 7.4 Hz, 4H), 7.65–7.55 (m, 6H), 7.51–7.45 (m, 2H), 7.43–7.37 (m, 2H), 7.07 (d, *J* = 9.1 Hz, 2H). ^13^C NMR (101 MHz, DMSO) *δ* 178.04, 165.74, 156.52, 152.69, 145.11, 142.51, 133.38, 131.26, 128.61, 122.99, 122.28, 121.28, 120.10, 119.20, 111.13, 109.96

Correspondingly, ^13^C NMR data (summarized in the experimental section and Table 3) also corroborated well with the formation of ligands L_1_ and L_2_ (Fig. S8a and S9b†). All assignments of the carbon atoms were found in their expected regions^29^ and well supported by their IR and ^1^H NMR spectral data. The azomethine and phenolic carbon of ligands L_1_ and L_2_ shifted downfield in complexes, *i.e.* from *δ* 172 (L_2_ and Fig. S9b†) to *δ* 182 (complex 7 and Fig. S11b†), indicating the involvement of azomethine-*N* and phenolic-*O* in coordination with the metal ion. Furthermore, the aromatic carbons found near the coordination sites showed a slight downfield shift (Table 3, Fig. S6b–S8b and S10b–S12b†), indicating the shift of electron density from ligand donor sites to the metal ion.^30^

### 2.3 Mass spectral studies

**Fig. 2:**
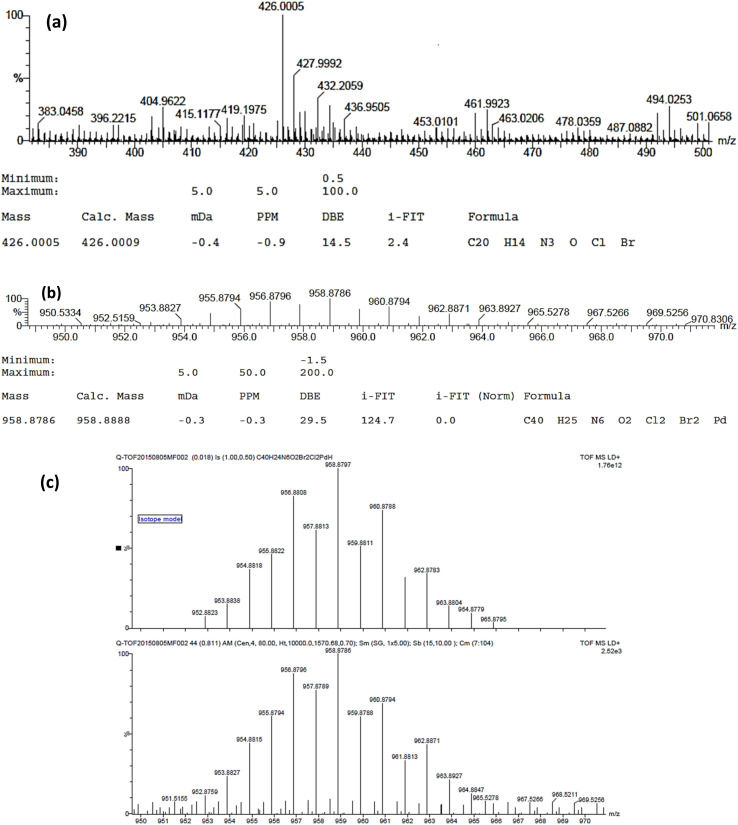
Representative high-resolution ESI mass spectrum of ligand (L_1_) (a), Pd(ii) complex (3) (b), and isotope model of Pd(ii) complex (3) (c).

The molecular weight and stoichiometric composition of the ligands and complexes were determined using the respective *m*/*z* values in the mass spectrum of the compounds. The successful synthesis of ligands L_1_ and L_2_ was proved by the presence of base peak signals at *m*/*z* = 426 (M + H) and 364 (M + H), respectively (Fig. 2a and S14†). The molecular ion peaks at *m*/*z* = 916 (M + H^+^), 910 (M + H^+^), 959 (M + H^+^), 917 (M + H^+^), 789 (M + H^+^), 786 (M + H), 831 (M + H^+^) and 792 (M + H) confirmed the molecular weight of complexes 1–8, respectively, as shown in Fig. 2b and c and S15–S21 in the ESI.†

### 2.4 Single-crystal X-ray diffraction analysis of L_1_

**Fig. 3:**
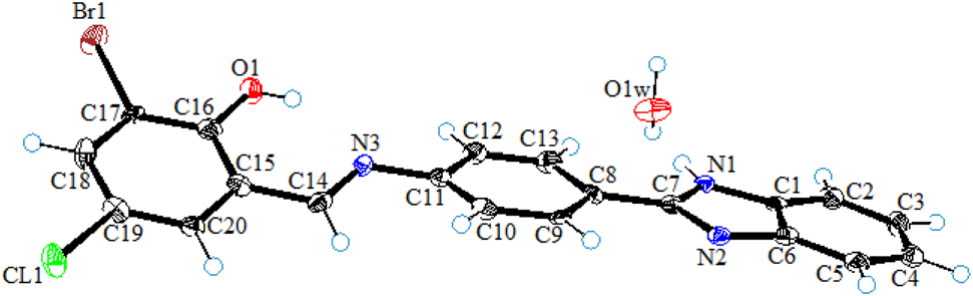
The ORTEP view of ligand (L_1_) drawn at a 50% probability level.

Single-crystal X-ray diffraction studies of ligand (L_1_) was carried out by mounting an orange color crystal of suitable size 0.02 × 0.09 × 0.30 mm^3^ on a narrow glass fiber fixed with wax on the copper pin for data collection. The collection of data was carried out using a Bruker D8 venture fitted with a CCD detector diffractometer and graphite monochromator with Cu K_α_ radiation (*λ* = 1.54178 Å) at *T* = 100(2) K. The integration and reduction of the collected data were done using the SAINT program. The structure was solved by applying the direct method and Fourier transformation techniques, and further refinement was performed using the SHELXL97 program with full-matrix least-square calculations on *F*^2^. PLATON and SHELXL programs were used for the final refinement of the structure. The ORTEP view in Fig. 3 represents the crystal structure of ligand (L_1_) and the MERCURY program used for drawing crystal packing and intermolecular interactions present in the crystal lattice.

A summary of crystal data collection and refinement is presented in Table 4.

**Table 4:** Crystal data and structure refinement for L_1_

Empirical formula	C_40_H_30_Br_2_Cl_2_N_6_O_4_
Formula weight	889.42
Temperature	100(2) K
Wavelength	1.54178 Å
Crystal system	Orthorhombic
Space group	*P*2_1_2_1_2_1_
Unit cell dimensions	*a* = 8.3713(3) Å, *a* = 90°
	*b* = 14.0145(4) Å, *b* = 90°
	*c* = 31.3792(9) Å, *g* = 90°
Volume	3681.4(2) Å^3^
*Z*	4
Density (calculated)	1.605 Mg m^−3^
Absorption coefficient	4.558 mm^−1^
*F*(000)	1792
Crystal size	0.300 × 0.090 × 0.020 mm^3^
Theta range for data collection	3.454 to 66.683°
Index ranges	−9 ≤ *h* ≤ 9, −16 ≤ *k* ≤ 16, −33 ≤ *l* ≤ 37
Reflections collected	25 443
Independent reflections	6474 [*R*(int) = 0.0913]
Completeness to theta = 66.683°	99.8%
Refinement method	Full-matrix least-squares on *F*^2^
Data/restraints/parameters	6474/1/512
Goodness-of-fit on *F*^2^	1.028
Final *R* indices [*I* > 2sigma(*I*)]	*R*_1_ = 0.0406, w*R*_2_ = 0.0762
*R* indices (all data)	*R*_1_ = 0.0542, w*R*_2_ = 0.0805
Absolute structure parameter	0.47(2)
Extinction coefficient	n/a
Largest diff. peak and hole	0.339 and −0.398 e Å^−3^

#### 2.4.1 Structural features

The asymmetric unit of ligand (L_1_) comprises two independent water molecules, each as water hydrate. Structurally, ligand (L_1_) was found to be composed of a central planner benzene ring (C8–C13) attached with benzimidazole moiety (C7/N1–N2/C1–C6) at C8 and iminomethyl-6-bromo-4-chlorophenol (N3/C14–C20/CL1/Br1/O1) ring at C11. The dihedral angles between the plane of a central benzene ring with iminomethyl-6-bromo-4-chlorophenol (N3/C14–C20/CL1/Br1/O1) and benzimidazole moieties (C7/N1–N2/C1–C6) were found to be 22.526° and 10.345°, respectively, while in molecule 2, these angles were 22.287° and 3.375°, respectively. The difference between the angles of the rings of molecule 1 and molecule 2 leads to their independent appearance in the asymmetric unit.

#### 2.4.2 Crystal packing

**Fig. 4:**
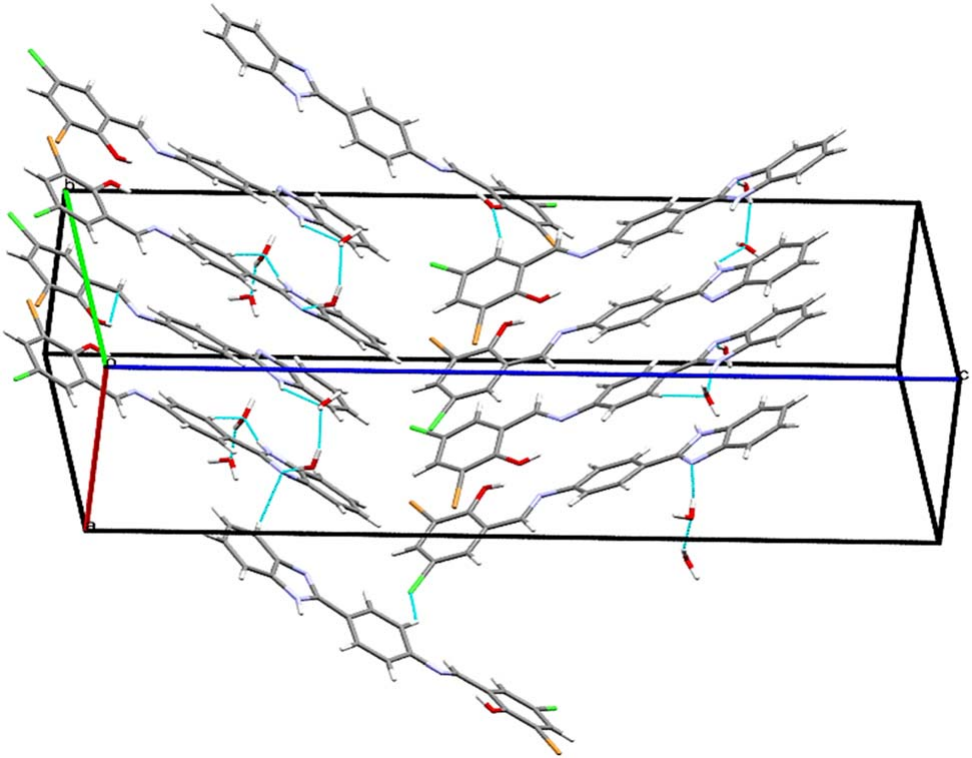
Crystal packing diagram of ligand (L_1_) showing intermolecular interactions within the unit cell along the *b*-axis.

In the crystal lattice, molecules are found to be connected *via* H1A⋯O1W, H1W⋯N2, H3W⋯O1W, H4W⋯N4, H5A⋯O2W, H5⋯N4, H10⋯CL1, H29⋯O2W, and H40⋯O1 intermolecular interactions with the donor–acceptor distance of 2.7810 Å, 2.6900 Å, 2.7711 Å, 2.8130 Å, 2.7473 Å, 3.4290 Å, 3.5941 Å, 3.3823 Å, and 3.3375 Å, respectively, to form a 3D network as shown in Fig. 4. A list of selected hydrogen bonds geometry in the ligand (L_1_) is given in Table 5.

**Table 5:** List of selected hydrogen bond geometry (Å) in the ligand (L_1_)^a^

D	H	A	D–H	H⋯A	D⋯A	D–H⋯A
N(1)	H(1A)	O(1W)	0.88	2.05	2.7810(1)	140
O(1W)	H(1W)	N(2)	0.92	1.77	2.6900(1)	171
O(2W)	H(3W)	O(1W)	0.78	2.02	2.7711(1)	161
O(2W)	H(4W)	N4	0.89	1.93	2.8130(1)	168
N5	H(5A)	O(2W)	0.88	1.88	2.7473(1)	170
C(5)	H(5)	N(4)	0.95	2.61	3.4290(1)	145
C(10)	H(10)	CL(1)	0.95	2.82	3.5941(1)	140
C29	H(29)	O(2W)	0.95	2.46	3.3823(1)	165
C40	H(40)	O(1)	0.95	2.45	3.3375(1)	156

a Symmetric codes: 1/2 − *X*, −*Y*, 1/2 + *Z*, −*X*, 1/2 + *Y*, 1/2 − *Z*, 1/2 + *X*, 1/2 − *Y*, and −*Z*.

### 2.5 DNA binding studies

The DNA binding proclivity of the synthesized compounds was investigated theoretically by molecular docking and experimentally by UV-vis absorption titrations and thermal denaturation studies, as illustrated below.

#### 2.5.1 Molecular docking studies

**Fig. 5:**
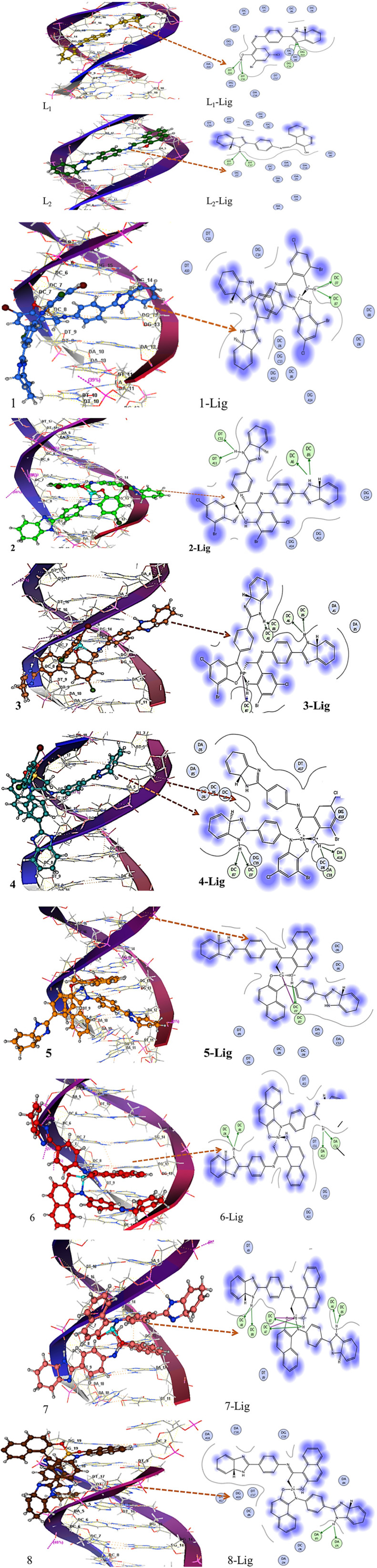
3D complex (left) and 2D lig plot (right) showing docking interaction map of ligands L_1_ and L_2_ and representative complexes 2–8. Hydrogen bonding is shown by the orange dotted line.

Molecular docking is the most suitable technique that provides a theoretical approach for structure-based drug design.^31,32^ A theoretical study preliminary declares the compound a potential drug candidate for passing through experimental trials.^33^ Thus, theoretical studies using molecular docking were carried out for the binding and possible interaction of ligands (L_1_ and L_2_) and their complexes with DNA before proceeding to the experimental investigations. A 3D pose view analysis of the lowest energy conformation of ligands (L_1_ and L_2_) and complexes of ligands (1–8) is shown in Fig. 5 (left). The docked conformation of compounds indicates that L_1_, L_2_ and their coordination complexes interacted with DNA *via* intercalation and groove bonding mode. However, intercalation can be more profoundly observed in complexes 3, 4, 5, 7 and 8 owing to the sliding of the planar part of the structure into stacked DNA base pairs. Ligplots revealing the 2D profile for the interaction of DNA base pair residues are depicted in Fig. 5 (right). Blue blurred regions in the ligplots represent the direct exposure of compounds to solvents. It was observed from the 3D docked images (Fig. 5 (left)) and corresponding 2D ligplots that all the compounds, entailing ligands and their respective complexes, furnished H-bonding with the base pairs of DNA. It is clear that ligand L_1_ developed two hydrogen bonds between the phenolic-H with a C=O oxygen of thymine DT-C16 and DT-A16, whereas two hydrogen bonds were built between the N atom of L_1_ and the H atom of cuanine and cytosine, respectively, *i.e.*, DG-A14 and DC-G14 of DNA. The 3D and 2D pose images of L_1_–Ni complex 2 (Fig. 5 (3)) indicated the formation of four hydrogen bonds with –NH_2_ of cytosine (DC-A6 and DC-B8) as well as with a C=O group of thiamine (DT-C11, DT-A11) of DNA base pairs. Compound 2 showed a greater DNA binding propensity owing to its capacity to develop a greater number of direct hydrogen bonds with nitrogenous bases of DNA. Thus, the docked results suggest an intercalative mode and groove binding of the studied compounds. Complex 1 was able to develop two hydrogen bonds with an –NH_2_ group of DC-D7 and DC-B7 DNA, suggesting a comparatively smaller value of binding for complex 1.

Similar docking simulations were carried out for ligand L_2_ and metal complexes 5–8. From the 3D pose and 2D ligplot images (Fig. 5), it can be observed that ligand L_2_ formed two hydrogen bonds involving phenolic-H and the imidazole ring nitrogen atom as a binding site. The most effective binding interactions were shown by the Pd complex of L_2_ with the following six hydrogen bonds: DC-A8, DC-B8, DC-A7, DC-B7, DC-A6, and DC-B6. Complex 8 exhibited minimum binding, as indicated in Fig. 5 (right). It exhibited only two hydrogen bonds, which were formed with an –NH_2_ group of adenine DA-B5 and DA-D5. The intercalation of the planar aromatic part of L_2_ complexes is also evident along with the mixed mode of interactions.

#### 2.5.2 UV-visible absorption spectroscopy

Electronic absorption spectroscopy is an important qualitative and quantitative technique used for DNA binding investigations. The information regarding binding interactions between a compound and DNA can be collected by measuring the changes in the absorption spectra of the compound alone and compound–DNA mixture. It entails a shift in absorbance (hypochromism or hyperchromism) and a shift in *λ*_max_ (blue or red shift).

**Fig. 6:**
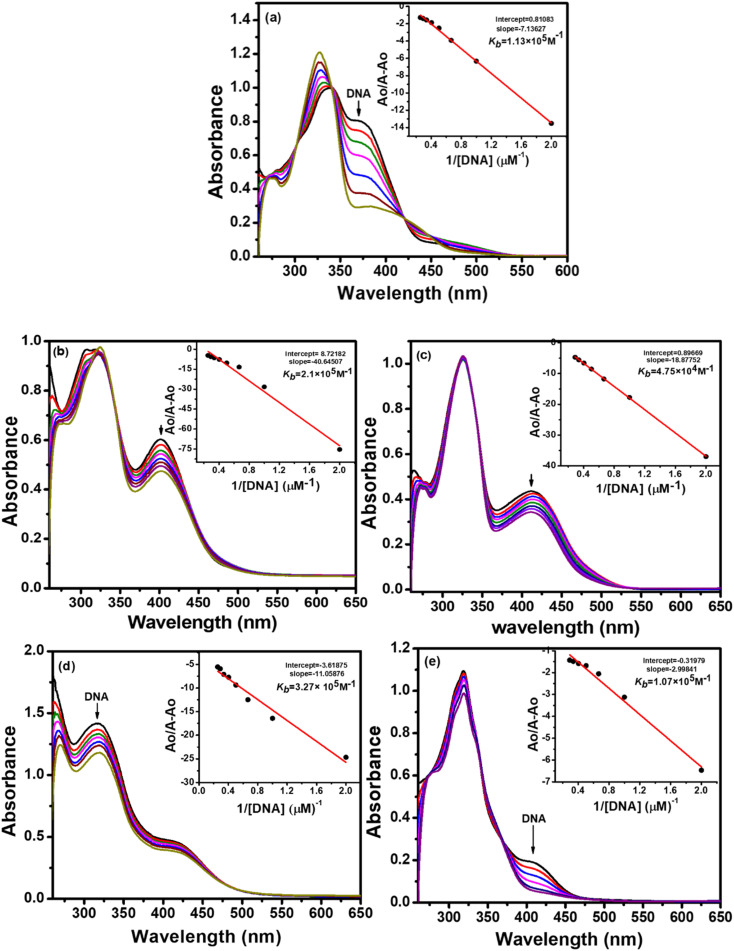
Changes in the UV-vis spectra of ligand L_1_ (a), Cu(ii) complex 1 (b), Ni(ii) complex 2 (c), Pd(ii) complex 3 (d), and Zn(ii) complex 4 (e), having a concentration of 1 × 10^−4^ M, as a function of increasing quantities of fish sperm DNA (10–80 μM) in 50 mM Tris–HCl/NaCl buffer (pH = 7.35) at 25 °C. The arrows indicate a decrease in absorbance with an increase in DNA concentration. Inset plots show intrinsic binding constant *k*_b_.

**Fig. 7:**
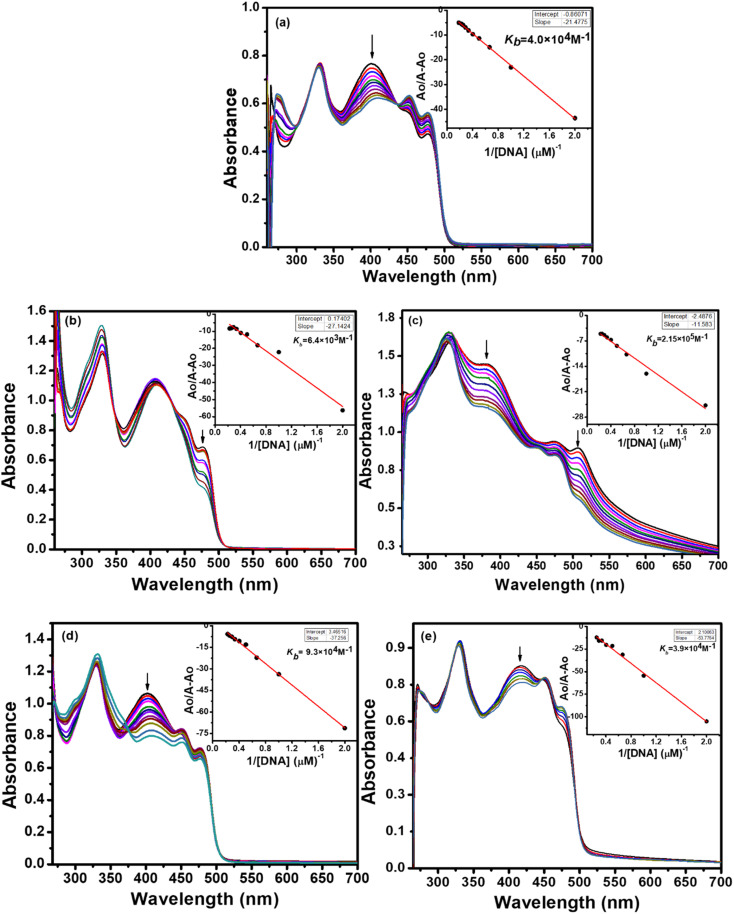
Changes in the UV-vis spectra of ligand L_2_ (a), Cu(ii) complex 5 (b), Ni(ii) complex 6 (c), Pd(ii) complex 7 (d) and Zn(ii) complex 8 (e) having a concentration of 1 × 10^−4^ M, as a function of increasing quantities of fish sperm DNA (10–80 μM) in 50 mM Tris–HCl/NaCl buffer (pH = 7.35) at 25 °C. The arrows indicate a decrease in absorbance with an increase in DNA concentration. Inset plots show intrinsic binding constant *k*_b_.

The absorption spectra resulting from titrations are depicted in Fig. 6 and 7. As observed from spectral curves, the absorbance of test compounds decreased with the addition of SS-DNA aliquots (10–80 μM). This hypochromism accompanied by a red shift/blue shift (Table 7) suggests that these compounds bind to DNA through intercalation mode and groove binding.^34,35^ The hypochromic effect is considered to be due to the interaction between the electronic states of the intercalating chromophore (compounds) and the nitrogen bases of DNA.^36^ This resulted in structural changes in DNA that entailed the local unwinding of the double helix and the creation of a cavity to accommodate guest compound molecules.^37^ It is also obvious from previous knowledge that the electronic interaction between chromophores and DNA bases decreases as a cube of the distance of separation between them.^35^ The maximum hypochromism was observed in our case for compound 2, which was 73%, indicating the very close proximity of compound 2 chromophores to DNA bases. The other compounds, including ligands and complexes, showed hypochromism from 28–57%, suggesting their stronger binding interaction with DNA (Table 7).

To quantitatively compare the binding strength of the ligands and complexes with SS-DNA, the intrinsic binding constants (*K*_b_) were calculated from the absorption data; their values are collated in Table 7. In the first series (L_1_ and its complexes), the highest binding constant was observed for Ni(ii) complex 2 and the lowest for Pd(ii) complex 3. By comparing the calculated binding constant values of test compounds with that of the known intercalator, proflavine,^38^ it was inferred that compounds 1 and 2 were stronger intercalators, while compounds L_1_, 3, and 4 bind to DNA *via* partial intercalation along with other modes, such as groove binding. This type of interaction of compounds 1 and 2 could be justified by the square planar geometry of Ni(ii) and Cu(ii) complexes (confirmed by the diamagnetic nature of Ni(ii) and the d^9^ electronic configuration of Cu(ii)^39^), facilitating them to diffuse between the DNA bases more effectively; hence, stronger stacking interactions are developed.

In the second series (L_2_ and complexes), the highest binding constant was observed for Ni(ii) complex 6 and the lowest for Cu(ii) complex 5. Thus, compound 6 interacted with DNA through intercalation, while compounds L_2_, 5, 7 and 8 interacted through partial intercalation and groove binding. The trend in the hypochromism between the ligands followed the order L_1_ > L_2_. The same trend was found with the binding constant values. Thus, the two results supported each other. The trend in hypochromism among complexes was of the following order: 2 > 6 > 1 > 4 > 7 > 3 > 8 > 5. The same order was found for the binding constant values, as illustrated in Table 7. Thus, the magnitude of the hypochromism reflects the strength of the stacking interactions between the compound and DNA.

In summary, among the synthesized compounds, Ni(ii) complex 2 exhibited the strongest interaction with DNA. The corresponding absorption spectrum showed an isobestic point at 344 nm, indicating DNA-2 complex formation and a hypochromic effect along with bathochromic shift Δ*λ* = 4 nm. Similarly, complexes 6 and 1 also manifested stronger DNA interaction proclivity (see Table 6). Hence, it was concluded that complexes 2, 6, and 1 bind with DNA through intercalation, and the rest of the compounds interact through partial intercalation and groove binding.

**Table 6:** Binding constants (*K*_b_) and free energy (Δ*G*) for the interaction of DNA with ligands L_1_, L_2_, and metal complexes (1–8) calculated from molecular docking data

Compound	*K*_b_ (M^−1^)	Δ*G* (kJ mol^−1^)
L_1_	1.55 × 10^5^	−29.68
L_2_	5.11 × 10^4^	−26.88
1	2.33 × 10^5^	−30.62
2	45.8 × 10^5^	−38.01
3	7.46 × 10^4^	−27.80
4	2.34 × 10^5^	−30.64
5	1.14 × 10^4^	−23.16
6	1.52 × 10^5^	−29.57
7	2.85 × 10^5^	−31.12
8	6.56 × 10^4^	−27.48

It is established from the results of hypochromicity and intrinsic binding constant values that the synthesized compounds show binding interactions with DNA. Further experiments must be conducted to determine the energy parameters for compound–DNA complexes.

The Gibb's free energy (Δ*G*) of the compound–DNA complex explained the thermodynamic stability of the compound–DNA complex when DNA was added to the solution of the compound. Eqn (1) helps to calculate free energy by putting the values of suitable parameters:

1 Δ*G* = −*RT*ln *K*_b_.

A comparison of the free energy values (Table 7) indicates that the binding of Ni(ii) complex 2 with DNA was most spontaneous and thermodynamically favorable under the given set of conditions. The remaining compounds also generated stable adducts with DNA and could be used as future therapeutic agents to cure various disorders in the living system.

**Table 7:** Increments on (st) DNA thermal denaturation (Δ*T*_m_, °C), binding constants (*K*_b_, M^−1^), hypochromism, Δ*λ*_max_ and isobestic points (nm) found for ligands L_1_, L_2_ and metal complexes 1–8

Compound	Δ*T*_m_^a^ (°C)	*K*_b_ b (M^−1^)	Hypochromism (%)	Δ*λ*_max_	Δ*G* (kJ mol^−1^)	Isobestic points (nm)
L_1_	4.2	1.13 × 10^5^	43	15 nm (red)	−28.81	301, 341, 422
L_2_	3.1	4.00 × 10^4^	35	9 nm (red)	−26.24	438
1	5.7	2.1 × 10^5^	60	2 nm (blue)	−30.34	342
2	6.1	3.27 × 10^5^	75	4 nm (blue)	−31.44	344
3	2.7	4.75 × 10^4^	39	3 nm (red)	−26.66	—
4	3.6	1.07 × 10^5^	55	8 nm (red)	−28.67	338, 371
5	2.2	6.40 × 10^3^	28	2 nm (red)	−21.70	344, 432
6	4.7	2.15 × 10^5^	73	7 nm (blue)	−30.40	452
7	3.2	9.3 × 10^4^	42	6 nm (red)	−28.33	376
8	3.9	3.9 × 10^4^	35	4 nm (blue)	−26.18	444, 464

a The increment in DNA thermal melting (Δ*T*_m_, °C) was measured in unspecific (st) DNA. Experiments were run in phosphate buffer (10 mM, pH 7). Thermal denaturation for natural (st) DNA = 68.5 °C.

b Calculated from the *A*_0_/*A* − *A*_0_*vs.* 1/[DNA] plots of the UV-vis DNA titration results.

#### 2.5.3 Thermal denaturation study

DNA melting study provided stronger evidence for the interaction of compounds with double helix.^40^ The absorption spectra of nitrogenous bases (*λ*_max_ = 260 nm) showed a hyperchromic effect when the double-stranded structure dissociated into single strands as the temperature of the solution increased. The temperature at which 50% of the molecules underwent this transition was called the melting temperature “*T*_m_”.^41,42^ The literature revealed that the intercalation of compounds stabilizes the double helix structure of DNA by stacking interactions, resulting in an increase in *T*_m_.^43–45^

**Fig. 8:**
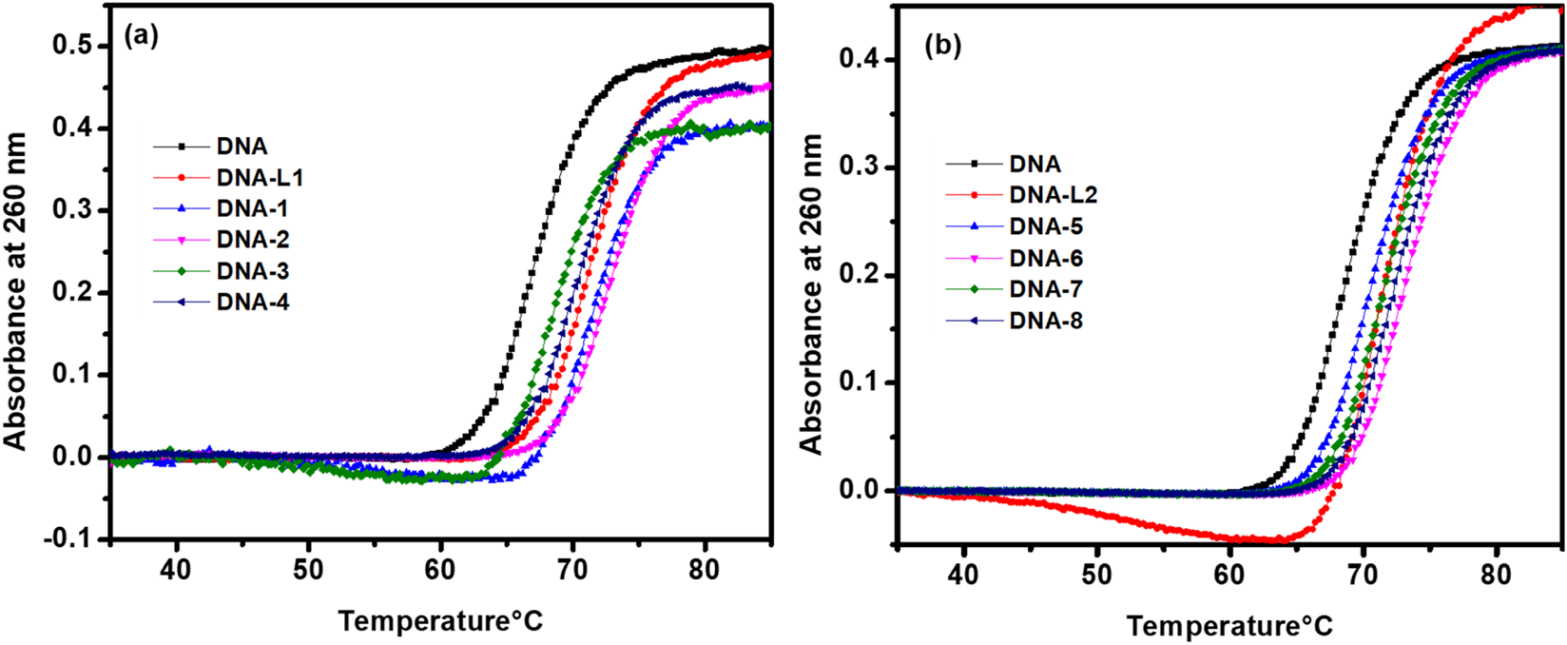
Melting curves for 100 μM of ST-DNA upon the addition of ligand L_1_ and complexes 1–4 (a), ligand L_2_ and complexes 5–8 (b).

The transition in the structure of DNA from double-stranded to single strands was monitored by measuring absorbance at 260 nm as a function of temperature. Fig. 8a and b indicate that in the absence of any compound, the *T*_m_ of ST-DNA was 68.5 °C under our experimental conditions. The melting point increased in the range of 71.2 to 74.6 °C for compounds of the first series (ligand L_1_ and complexes 1–4) and 70.7 to 73.2 °C for the second series (L_2_ and complexes 5–8), as indicated in Table 7. From the results, it was deduced that all compounds interacted efficiently with DNA *via* an intercalation mode. This type of stacking interaction stabilizes the double helix structure of DNA and increases *T*_m_. The significantly increased *T*_m_ of DNA in the presence of complexes 2 and 6 revealed a very strong interaction between the compound and DNA, which was also reflected in the binding constant (*K*_b_) values obtained spectroscopically.

### 2.6 Biochemistry

#### 2.6.1 Antibacterial activities

The antibacterial activities of ligands and their metal complexes against two strains of Gram-positive *M. luteus* (ATCC 10240), *S. aureus* (ATCC 6538), and Gram-negative bacteria *E. coli* (ATCC 15224), and *E. aerogenes* (ATCC 13048) are listed in Table 8. The ligands L_1_ and L_2_ did not show any activity against *M. luteus* and *S. aureus*, but they showed moderate activity against *E. coli* and *E. aerogenes*. Among the metal complexes, only Ni(ii) complexes 2 and 6 showed activity against all strains of bacteria. The obtained results indicated that metal complexes exhibited higher antibacterial activity compared to their respective ligands for the same bacterial strain under similar experimental conditions. Thus, it was suggested that chelation could facilitate the permeation of a compound across the outer cell barrier according to Tweedy's theory. In a complex, the positive charge of the metal ion is considerably reduced mainly because of the partial sharing of its positive charge with electron donor groups of ligands and by the delocalization of pi electrons on the aromatic rings. Such chelation could enhance the lipophilic character of the complex and favor its permeation through the lipid layer of the cell membrane, interact with DNA, and initiate the death of microorganisms.^46^ The difference in the activity of compounds depends either on variations in the permeability of the microbial lipid cell membrane or on differences in the ribosomes of bacterial cells.^47^ The activity of the antibiotic “kanamycin” (21.2–26.3 mm) against various bacterial strains relative to the activity of test compounds (4.7–16.8 mm) suggested that the antibacterial effect of the latter was slightly lower and Ni(ii) complexes 2 and 6 displayed broad-spectrum antibacterial activity against all strains used in the experiment similar to that of antibiotics, although with lower magnitude. Minimum inhibitory concentration (MIC) was also calculated as an index of antimicrobial activity by downstream calculations for those compounds that showed activity at 200 μg mL^−1^ by making working dilutions (200, 100, 50, 25, 12.5, and 6.25 μg mL^−1^), as illustrated in Table S1 of ESI.† Ni(ii) complexes 2 and 6 were the outcomes of research that could be moderately broad-spectrum antibiotics at low concentrations; by further optimization, the targeted results can be achieved. The transition metals used here are the essential bioelements present in the living organism at various concentrations.^12^ At higher concentrations, they are very toxic, but their low concentrations are necessary for proper body functions. Most organic molecules need metal ions for their activation and biotransformation, including metalloenzymes.^48^ Therefore, at tolerable concentrations, the Schiff base transition metal complexes can substitute for the already available antibiotics with severe side effects.

#### 2.6.2 Cytotoxicity and cell viability

The synthesized compounds were assessed for their preliminary cytotoxic activity by performing a brine shrimp lethality assay at three concentrations. Each test was carried out in triplicate with DMSO serving as a negative control and doxorubicin (LD_50_ = 3.2 μg mL^−1^) serving as a positive control. As an index of cytotoxicity, the LD_50_ values were calculated and collated, as presented in Table 9. The results indicated that most of the compounds were non-toxic towards normal shrimp cells with LD_50_ > 200 μg mL^−1^. Only two compounds, L_1_ (LD_50_ 127 μg mL^−1^) and 7 (LD_50_ 125 μg mL^−1^), showed slightly toxic behavior. After examining these findings, it is suggested that the synthesized compounds were non-toxic against normal shrimp cells. The reported cytotoxicity values of Cu(ii) (LD_50_ = 9.5 μg mL^−1^), Ni(ii) (LD_50_ = 15.6 μg mL^−1^) and Zn(ii) (LD_50_ = 17.8 μg mL^−1^)^49^ indicated the toxic nature of metals for shrimp cells. However, the toxic effect decreased considerably on the chelation of these metals with benzimidazole Schiff base ligands expectedly due to the increased lipophilic character of metal complexes. This activity relationship directs the development of future non-toxic antimicrobial agents for clinical applications.

**Table 8:** Antibacterial activities against *M. luteus*, *S. aureus*, *E. coli*, and *E. aerogenes*: inhibition zone (mm) at 200 μg mL^−1^ and MIC (μg mL^−1^) values

Compounds	Inhibition zone (mm)				MIC values (μg mL^−1^)			
	*M. luteus*	*S. aureus*	*E. coli*	*E. aerogenes*	*M. luteus*	*S. aurus*	*E. coli*	*E. aerogenes*
L_1_	—	—	7.1 ± 0.3	6.3 ± 0.5	—	—	100 ± 3.1	100 ± 2.5
L_2_	—	—	8.5 ± 0.5	5.2 ± 1.25	—	—	100 ± 2.02	100 ± 3.25
1	—	4.7 ± 1.03	13.1 ± 1.04	6.4 ± 0.4	—	100 ± 3.46	50 ± 0.5	100 ± 2.63
2	8.5 ± 0.4	10.3 ± 0.7	16.8 ± 2.7	14.5 ± 0.8	50 ± 3.5	25 ± 1.37	12.5 ± 0.5	25 ± 0.5
3	—	—	7.4 ± 0.5	—	—	—	100 ± 2.73	—
4	6.6 ± 0.5	—	11.4 ± 1.4	8.5 ± 1.52	100 ± 4.51	—	50 ± 1.41	50 ± 1.50
5	—	—	—	—	—	—	—	—
6	9.4 ± 0.3	7.20 ± 0.5	12.5 ± 1.6	6.5 ± 0.3	50 ± 2.1	100 ± 4.25	25 ± 0.5	100 ± 3.85
7	—	—	8.5 ± 0.9	6.8 ± 0.6	—	—	100 ± 2.5	100 ± 4.5
8	7.7 ± 0.5	—	10.8 ± 0.5	—	100 ± 4.07	—	50 ± 1.05	—
K a	24.6 ± 0.1	26.3 ± 0.3	21.2 ± 01	22.4 ± 0.2	12.5 ± 0.5	6.25 ± 0.3	6.25 ± 0.5	12.5 ± 0.75

a Kanamycin.

**Table 9:** Results of brine shrimp cytotoxicity of ligands and complexes^a^

Compounds	Brine shrimp cytotoxicity assay (LD_50_ = μg mL^−1^)
L_1_	127.61 ± 1.32
L2	>200
1	>200
2	>200
3	>200
4	—
5	—
6	>200
7	125 ± 2.43
8	—
Doxorubicin	3.22 ± 4.32

a Note: — represents inactivity.

#### 2.6.3 Cell viability

**Fig. 9:**
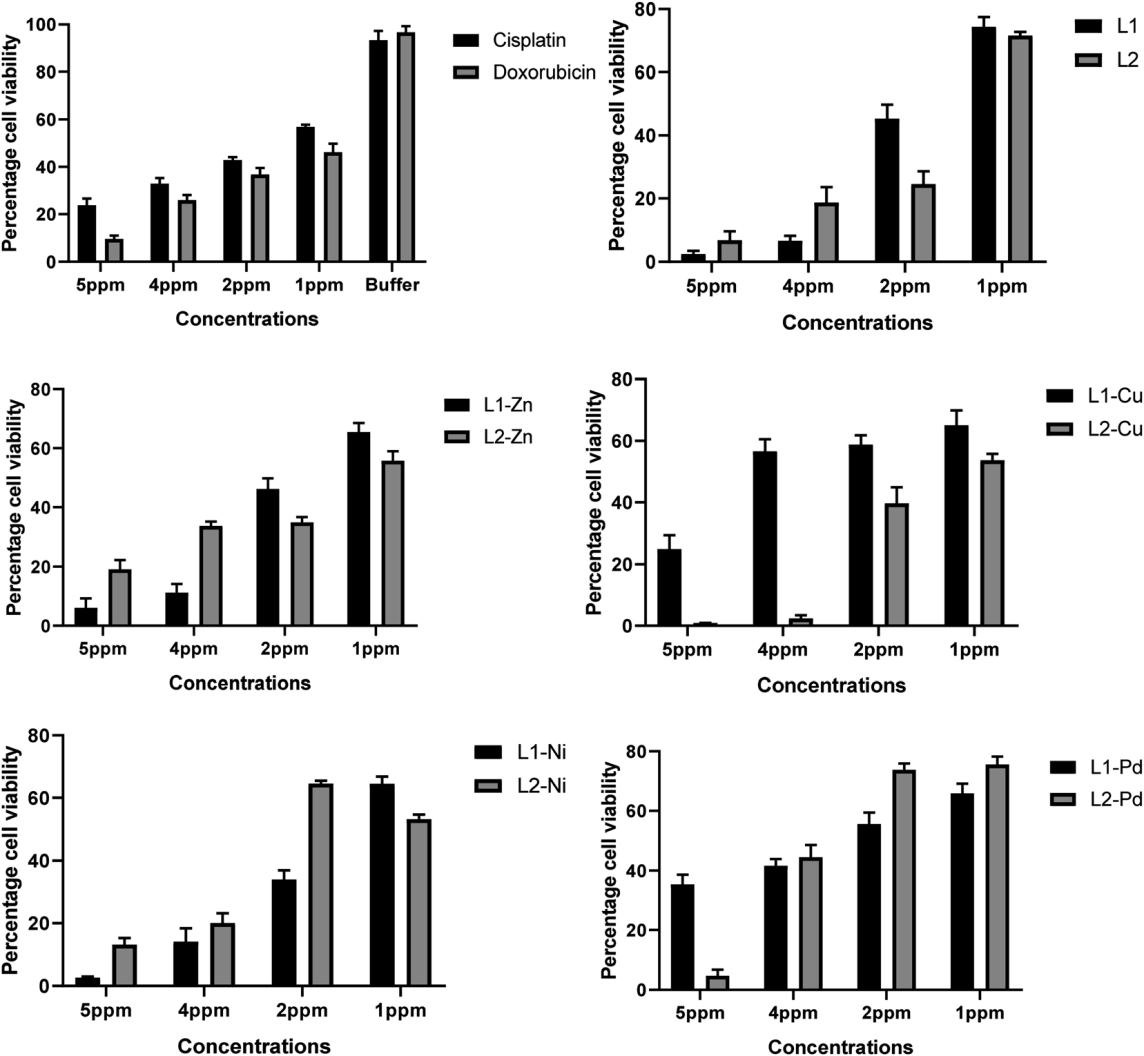
Percent cell viability of ligands L_1_ and L_2_ and their respective Zn(ii), Cu(ii), Ni(ii) and Pd(ii) complexes from 1 ppm to 5 ppm concentration against MCF7 cell line.

The cell viability of ligands L_1_ and L_2,_ and complexes 2–8 was tested against cancerous human breast cells (MCF7). For this purpose, various concentrations from 1 ppm to 5 ppm were used, and an ascending activity trend was observed. The study showed that the ligands and complexes were cytotoxic against cancer cells. The cytotoxic activity of ligands and complexes could be attributed to the benzimidazole N–H and azomethine (C=N) groups, respectively, which may be an important fragment for the formation of hydrogen bonding at the receptor site.^50,51^ Among these complexes, complexes 5 (L_2_-Cu), 2 (L_1_-Ni), and 7 (L_2_-Pd) exerted strong cytotoxic effects when compared with cisplatin and doxorubicin used as the standard, as illustrated in Fig. 9. This may partially be attributed to the lipophilic character of the central metal atom as explained based on Overton's concept and Tweedy's chelation theory.^52,53^ Complexes 5 (L_2_-Cu), 2 (L_1_-Ni), and 7 (L_2_-Pd) possess poor activity compared to those of the respective ligands, which could be attributed to their decreased solubility. A comparative study with cisplatin and doxorubicin based on % cell viability values obtained with the MCF7 cell line exhibited that the ligands L_1_, L_2_, and complexes 5 (L_2_-Cu), 2 (L_1_-Ni), and 7 (L_2_-Pd) showed percent cell viability values at 1.46, 4.12, 0.129, 1.89, and 3.09, respectively, while cisplatin and doxorubicin maintained 21.89 and 7.37 values correspondingly. The poor activity of Zn(ii) complexes is attributed to the restriction of only tetrahedral structure, while square planar complexes of remaining metal ions help in the effective penetration of complexes between the grooves of DNA and show better binding effects. The Ni(ii) and Pd(ii) complexes induce apoptosis (programmed cell death) that may be a direct result of their action on the cell, while Cu(ii) complexes on reduction to Cu(i) generate reactive oxygen species (ROS)^54^ such as hydroxyl radicals from H_2_O_2_ produced during normal cellular activities, leading to growth inhibition in tumor cells.^55^ The enhanced cytotoxic values of ligands L_1_ and L_2_ and complexes 5 (L_2_-Cu), 2 (L_1_-Ni), and 7 (L_2_-Pd) than cisplatin and doxorubicin support the use of the first-row transition metal complexes as effective anticancer agents, but their intrinsic side effects must be overcome before clinical trials.

## 3. Experimental

### 3.1 Materials and methods

All chemicals, including solvents and reactant materials, were purchased from Sigma Aldrich and were used without further purification. Salmon sperm DNA (st)DNA, was purchased from Sigma Aldrich (St Louis, MO, USA) with an extinction coefficient of *ε*_260_ = 6600 M^−1^ cm^−1^.

### 3.2 Instrumentation and measurements

Elemental analysis was performed using a CHNS932 (Leco-USA) elemental analyzer. Melting points were recorded using an MPD Mitamura Riken Kogyo (Japan) electrothermal apparatus. Infrared (IR) spectra were recorded using a PerkinElmer Spectrum One Spectrometer in a 4000–400 cm^−1^ region. ^1^H and ^13^C{^1^H} NMR spectra were obtained using a Bruker Avance 400 and Bruker DPX 400 and were referenced to the residual ^1^H and ^13^C resonances of the solvent used. High-resolution mass spectrometry analysis was carried out using a Premier Waters Maldi-quadrupole time-of-flight (Q-TOF) mass spectrometer equipped with Z-spray electrospray ionization (ESI) and matrix-assisted laser desorption ionization (MALDI) sources.

Single-crystal X-ray diffraction data were collected using Bruker APEX D8 Venture diffractometer fitted with PHOTON 100 detector (CMOS technology) and fine-focus sealed tube with an X-ray source [Cu Kα radiation, *α* = 1.54178 Å]. Reflection intensities were integrated using SAINT software. Absorption correction was done using the multi-scan method.

The structure was solved by direct methods using SHELXS97 (Sheldrick, 1997)^56^ and difmap synthesis using SHELXS96 (Sheldrick, 1996).^57^ All non-hydrogen atoms were refined anisotropically, and the hydrogen atoms were placed into the calculated positions.

### 3.3 Synthesis

**Scheme 1:**
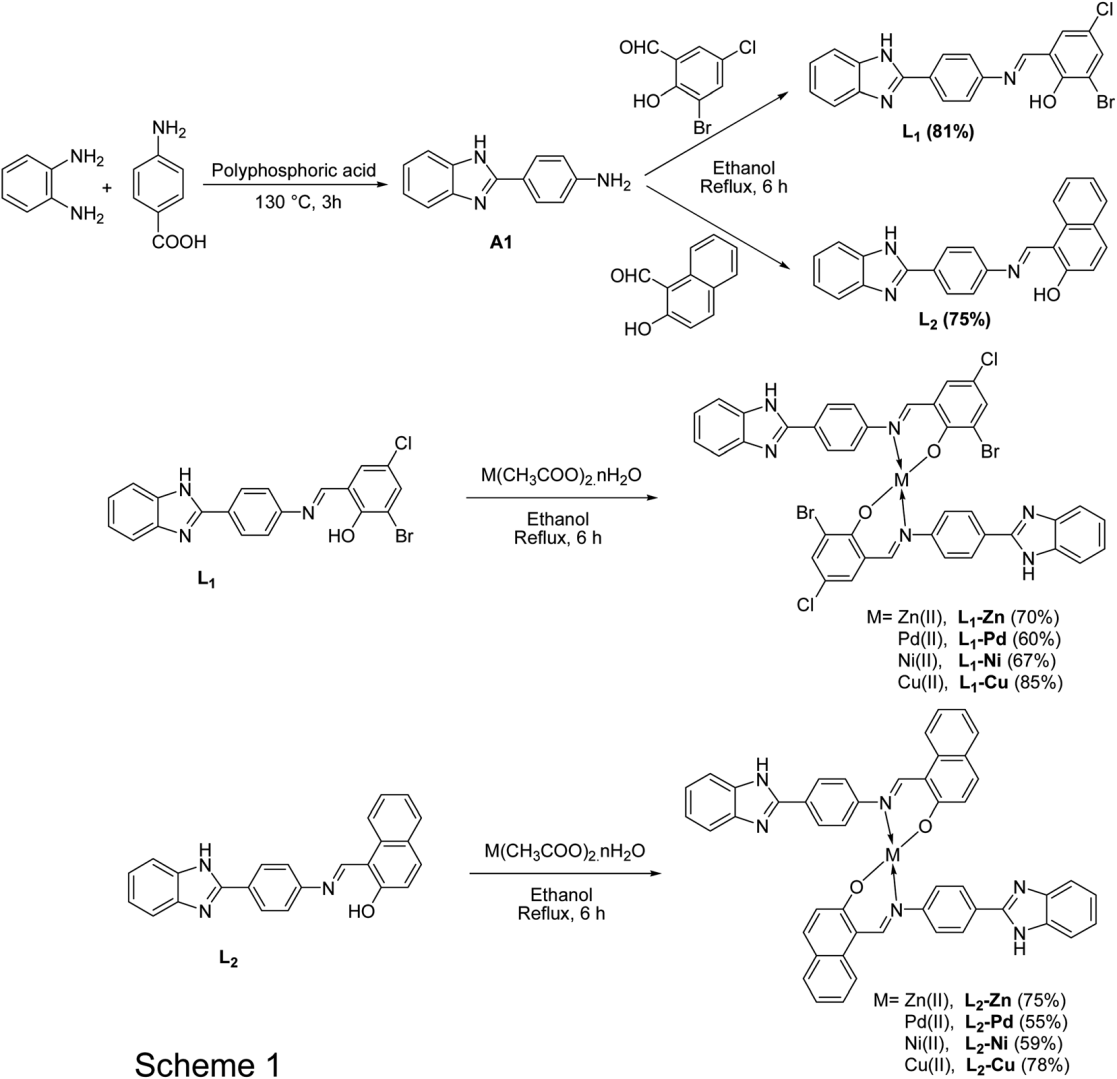
General synthetic chart of ligands and corresponding metal complexes.

The synthetic schemes for the ligands and corresponding metal complexes are shown in Scheme 1.

#### 3.3.1 Preparation of 4-(1*H*-benzo[*d*]imidazole-2-yl)aniline (A_1_)

In a two-neck round bottom flask (250 mL) equipped with a magnetic stirrer and reflux condenser, benzene-1,2-diamine and 4-aminobenzoic acid were refluxed in polyphosphoric acid in a 1 : 1 molar ratio. The reaction mixture was heated at 130 °C for 3 h. After completion of the reaction, the thick liquid mixture was poured onto crushed ice and neutralized with a 4 N NaOH solution, and the precipitate formed was filtered and dried. Compound A_1_ was extracted with methanol and used immediately for the preparation of L_1_ and L_2_.

#### 3.3.2 (*E*)-2-((4-(1*H*-benzo[*d*]imidazole-2-yl)phenylimino)methyl)-6-bromo-4-chlorophenol (L_1_)

(*E*)-2-((4-(1*H*-benzo[*d*]imidazole-2-yl)phenylimino)methyl)-6-bromo-4-chlorophenol was synthesized by reacting equimolar quantities of 4-(1*H*-benzo[*d*]imidazole-2-yl)benzenamine (A_1_) (0.21 g, 1 mmol) and 3-bromo-5-chloro-2-hydroxybenzaldehyde (0.23 g, 1 mmol) using dry ethanol under reflux for 6 h. Orange coloured precipitates of compound L_1_ were removed by filtration, washed with hot ethanol and recrystallized from ethanol. Yield: 86%. Colour: orange. M.p.: 174 °C. Anal. calc. (%) for C_20_H_13_N_3_OClBr: C, 56.52; H, 3.08; N, 9.88. Found: C, 56.38; H; 3.07; N, 9.86. IR (*ν*, cm^−1^): 3625 (N–H_ring_), 3295 (Br, O–H), 1619 (azomethine-C=N), 1434 (C–N_ring_). ^1^H NMR (400 MHz, DMSO) *δ* 14.49 (s, 1H), 13.02 (s, 1H), 9.13 (s, 1H), 8.35 (d, *J* = 8.5 Hz, 2H), 8.01 (d, *J* = 2.5 Hz, 1H), 7.95–7.78 (m, 2H), 7.62 (s, 1H), 7.49 (d, *J* = 3.2 Hz, 1H), 7.18 (ddd, *J* = 38.6, 5.8, 3.0 Hz, 2H), 6.67 (d, *J* = 8.6 Hz, 1H). ^13^C NMR (101 MHz, DMSO) *δ* 165.12, 158.81, 150.13, 142.31–140.17, 134.83, 129.02, 122.13, 119.79, 113.42, 110.82. HRMS (*m*/*z*) calculated for C_20_H_13_N_3_OClBr [M + H^+^] 426.0009, found 426.0005.

#### 3.3.3 (*E*)-1-((4-(1*H*-benzo[*d*]imidazole-2-yl)phenylimino)methyl)naphthalen-2-ol (L_2_)

(*E*)-1-((4-(1*H*-benzo[*d*]imidazole-2-yl)phenylimino)methyl)naphthalen-2-ol was synthesized by condensing equimolar quantities of 4-(1*H*-benzo[*d*]imidazole-2-yl)benzenamine (A_1_) (0.21 g, 1 mmol) and 2-hydroxy-1-naphthaldehyde (0.17 g, 1 mmol) according to the procedure discussed for the preparation of L_1_. Yield: 78%. Colour: red. M.p.: 169 °C. Anal. calc. (%) for C_24_H_17_N_3_O: C, 79.15; H, 4.70; N, 11.54. Found: C, 79.16; H; 4.73; N, 11.53. IR (*ν*, cm^−1^): 3618 (N–H_ring_), 3203 (Br, O–H), 1613 (azomethine-C=N), 1432 (C–N_ring_). ^1^H NMR (300 MHz, DMSO) *δ* 15.74 (s, 1H), 12.62 (s, 1H) 9.73 (s, 1H), 8.55 (d, *J* = 8.4 Hz, 1H), 8.28 (d, *J* = 8.4, 2H), 8.95 (d, *J* = 9, 1H), 7.85 (m, 3H), 7.42 (d, *J* = 10.5 Hz, 2H), 7.36 (d, m, 2H), 7.02 (d, *J* = 9 Hz, 2H). ^13^C NMR (101 MHz, DMSO) *δ* 171.98, 162.59, 155.67, 152.56, 145.34, 137.90 133.65, 129.54, 128.69, 128.18, 128.09, 127.16, 124.14, 121.43, 120.96, 110.59, 109.18. HRMS (*m*/*z*) calculated for C_24_H_17_N_3_O [M + H^+^] 364.1450, found 364.1459.

#### 3.3.4 Preparation of complexes of L_1_

An ethanolic solution of Schiff base L_1_ (2.5 mmol) was mixed with Cu(ii), Ni(ii), Pd(ii), and Zn(ii) metal ions using respective metal acetate (5 mmol) salt separately in a two-necked pre-backed round bottom flask. After stirring the whole mixture magnetically under reflux for 6 h, the coloured shining precipitate of the metal complexes formed was filtered, washed with ethanol, and dried over anhydrous CaCl_2_ in a vacuum desiccator.

##### 3.3.4.1 L_1_-Cu(ii) complex (1)

Yield: 83%. M.p.: >300 °C. Colour: brown. Anal. calc. (%) for C_40_H_24_N_6_O_2_CuCl_2_Br_2_ Calc. (%) C, 52.51; H, 2.64; N, 9.18. Found (%): C, 52.47; H, 2.63; N, 9.17. IR (*ν*, cm^−1^): 3620 (N–H_ring_), 1587 (azomethine, –C=N), 1447 (C–N_ring_), 569 (M–O), 490 (M–N). HRMS (*m*/*z*) calculated for C_40_H_24_N_6_O_2_CuCl_2_Br_2_ [M + H^+^] 914.9171, found 916.0054.

##### 3.3.4.2 L_1_-Ni(ii) complex (2)

Yield: 67%. Colour: red. M.p.: >300 °C. Anal. calc. (%) for C_40_H_24_N_6_O_2_Cl_2_NiBr_2_: C, 52.79; H, 2.65; N, 9.23. Found: C, 52.80; H, 2.68; N, 9.17. IR (*ν*, cm^−1^): 3650 (N–H_ring_), 1587 (azomethine, –C=N), 1442 (C–N_ring_), 562 (M–O), 476 (M–N). ^1^H NMR (400 MHz, DMSO) *δ* 14.52 (s, 1H), 9.52 (s, 1H), 8.24 (dd, *J* = 24.3, 6.1 Hz, 4H), 7.83 (t, *J* = 22.3 Hz, 2H), 7.65 (dd, *J* = 27.7, 18.9 Hz, 2H), 7.35 (s, 1H), 7.26 (s, 1H), 7.06 (s, 2H), 6.94 (d, *J* = 7.7 Hz, 1H), 6.65 (d, *J* = 8.6 Hz, 1H). ^13^C NMR (101 MHz, DMSO) *δ* 170.51, 159.91, 151.65, 148.61–146.26, 142.19, 138.96, 137.03, 121.91–110.52. HRMS (*m*/*z*) calculated for C_40_H_24_N_6_O_2_Cl_2_NiBr_2_ [M + H^+^] 910.1233, found 910.1239.

##### 3.3.4.3 L_1_-Pd(ii) complex (3)

Yield: 62%. Colour: brown. M.p.: >300 °C. Anal. calc. (%) for C_40_H_24_N_6_O_2_Cl_2_PdBr_2_: calc. (%): C, 50.10; H, 2.51; N, 8.77. Found (%): C, 50.07; H, 2.48; N, 8.76. IR (*ν*, cm^−1^): 3656 (N–H_ring_), 1598 (azomethine, –C=N), 1442 (C–N_ring_), 576 (M–O), 461 (M–N). ^1^H NMR (400 MHz, DMSO) *δ* 14.41 (s, 1H), 9.50 (s, 1H), 7.91 (d, *J* = 7.2 Hz, 4H), 7.73–7.65 (m, 6H), 7.51 (s, 2H), 7.05 (d, *J* = 7.1 Hz, 4H), 6.82 (d, *J* = 8.2, 4H). ^13^C NMR (101 MHz, DMSO) *δ* 166.15, 158.79, 151.75, 148.71, 147.19, 145.82, 144.36, 135.03, 128.61, 127.52, 123.58–11.15. HRMS (*m*/*z*) calculated for C_40_H_24_N_6_O_2_Cl_2_PdBr_2_ [M + H^+^] 958.88, found 958.8786.

##### 3.3.4.4 L_1_-Zn(ii) complex (4)

Yield: 77%. Colour: yellow. M.p.: >300 °C. Anal. calc. (%) for C_40_H_24_N_6_O_2_Cl_2_ZnBr_2_: C, 52.40; H, 2.63; N, 9.16. Found: C, 52.38; H, 2.58; N, 9.12. IR (*ν*, cm^−1^): 3615 (N–H_ring_), 1594 (azomethine, –C=N), 1429 (C–N_ring_), 576 (M–O), 426 (M–N). ^1^H NMR (400 MHz, DMSO) *δ* 14.47 (s, 2H), 9.46 (s, 2H), 8.30 (d, *J* = 7.2 Hz, 4H), 8.01–7.87 (m, 4H), 7.81 (s, 2H), 7.70 (d, *J* = 7.1 Hz, 4H), 7.62 (s, 2H), 7.23 (dd, *J* = 17.2, 14.5 Hz, 2H), 6.74 (d, *J* = 7.1 Hz, 2H). ^13^C NMR (101 MHz, DMSO) *δ* 169.21, 158.92, 151.42, 149.73, 146.11, 144.23, 138.82, 137.51, 135.02, 129.41, 128.12, 123.20–111.07. HRMS (*m*/*z*) calculated for C_40_H_24_N_6_O_2_Cl_2_ZnBr_2_ [M + H^+^] 916.9764, found 916.9791.

#### 3.3.5 Preparation of complexes of L_2_

Solutions of Schiff base ligand L_2_ (2.5 mmol) and acetates of Zn(ii), Pd(ii), Ni(ii), and Cu(ii) metal ions (5 mmol) were mixed in two necked pre backed round bottom flasks with constant stirring. After refluxing for 6 h, a colored shining precipitate of metal complexes formed was filtered, washed with ethanol, and dried over anhydrous CaCl_2_ in a vacuum desiccator.

##### 3.3.5.1 L_2_-Cu(ii) complex (5)

Yield: 75%. Colour: green. M.p.: 225 °C. Anal. calc. for C_48_H_32_N_6_O_2_Cu: Calc. (%): C, 73.03; H, 4.09; N, 10.65. Found (%): C, 72.98H, 4.05; N, 10.61. IR (*ν*, cm^−1^): 3622 (N–H_ring_), 1594 (azomethine, –C=N), 1446 (C–N_ring_), 540 (M–O), 433 (M–N). HRMS (*m*/*z*) calculated for C_48_H_32_N_6_O_2_Cu [M + H^+^] 789.2039, found 789.2026.

##### 3.3.5.2 L_2_-Ni(ii) complex (6)

Yield: 68%. Colour: dark red. M.p.: >300 °C. Anal. calc. for C_48_H_32_N_6_O_2_Ni: calc. (%): C, 73.35; H, 4.10; N, 10.69. Found (%): C, 73.37; H, 4.11; N, 10.71. IR (*ν*, cm^−1^): 3617 (N–H_ring_), 1587 (azomethine, –C=N), 1431 (C–N_ring_), 582 (M–O), 454 (M–N). ^1^H NMR (400 MHz, DMSO) *δ* 15.78 (s, 2H), 9.76 (s, 2H), 8.54 (s, 2H), 8.35–8.17 (m, 4H), 8.12 (d, *J* = 7.1 Hz, 2H), 7.95 (s, 2H), 7.83 (d, *J* = 22.4 Hz, 2H), 7.69 (d, *J* = 6.7 Hz, 4H), 7.63–7.49 (m, 6H), 7.38–7.10 (m, 4H), 7.10–6.83 (m, 2H). ^13^C NMR (101 MHz, CDCl_3_) *δ* 181.68, 164.21, 154.69, 150.07, 146.33, 144.0, 138.16, 132.30, 130.50, 129.50, 125.06, 124.03, 123.54–123.41, 122.03, 113.64–113.45, 110.02, 108.39. HRMS (*m*/*z*) calculated for C_48_H_32_N_6_O_2_Ni [M + H^+^] 785.9635, found 785.9643.

##### 3.3.5.3 L_2_-Pd(ii) complex (7)

Yield: 61%. Colour: brown. M.p.: >300 °C. Anal. calc. for C_48_H_32_N_6_O_2_Pd: calc. (%) C, 69.36; H, 3.88; N, 10.11. Found (%): C, 69.39; H, 3.89; N, 10.12. IR (*ν*, cm^−1^): 3615 (N–H_ring_), 1589 (azomethine, –C=N), 1429 (C–N_ring_), 554 (M–O), 433 (M–N). ^1^H NMR (400 MHz, DMSO) *δ* 15.91 (s, 2H), 10.03 (s, 2H), 8.58 (d, *J* = 8.8 Hz, 2H), 8.31 (s, 4H), 8.12 (m, 2H), 7.95 (dd, *J* = 34.4, 25.3 Hz, 2H), 7.84 (d, *J* = 7.4 Hz, 4H), 7.65 (d, *J* = 36.5 Hz, 6H), 7.52 (m, 2H), 7.40 (m, 2H), 7.07 (d, *J* = 9.1 Hz, 4H). ^13^C NMR (101 MHz, DMSO) *δ* 182.05, 169.15, 155.54, 150.72, 145.03, 137.90, 133.65, 129.54, 128.69–128.08, 127.14, 124.28–124.13, 122.91, 121.37, 109.14. HRMS (*m*/*z*) calculated for C_48_H_32_N_6_O_2_Pd [M + H^+^] 831.1700, found 831.1741.

##### 3.3.5.4 L_2_-Zn(ii) complex (8)

Yield: 79%. Colour: yellow. M.p.: 211 °C. Anal. calc. (%) for C_48_H_32_N_6_O_2_Zn: C, 72.78; H, 4.07; N, 10.61. Found (%): C, 72.72; H, 4.00; N, 10.55. IR (*ν*, cm^−1^): 3621 (N–H_ring_), 1594 (azomethine, –C=N), 1436 (C–N_ring_), 576 (M–O), 475 (M–N). ^1^H NMR (400 MHz, DMSO) *δ* 15.75 (s, 2H), 9.86 (s, 2H), 8.56 (d, *J* = 8.8 Hz, 2H), 8.32 (s, 4H), 8.13–8.10 (m, 2H), 8.05–7.95 (m, 2H), 7.84 (d, *J* = 7.4 Hz, 4H), 7.65–7.55 (m, 6H), 7.51–7.45 (m, 2H), 7.43–7.37 (m, 2H), 7.07 (d, *J* = 9.1 Hz, 2H). ^13^C NMR (101 MHz, DMSO) *δ* 178.04, 165.74, 156.52, 152.69, 145.11, 142.51, 133.38, 131.26, 128.61, 122.99, 122.28, 121.28, 120.10, 119.20, 111.13, 109.96. HRMS (*m*/*z*) calculated for C_48_H_32_N_6_O_2_Zn [M + H^+^] 792.0961, found 792.0940.

### 3.4 DNA binding studies

#### 3.4.1 Theoretical procedure

Molecular structures of ligands L_1_ and L_2_ and complexes (1–8) were built using the MOE.2015 window, optimized, and entered into molecular database entries. A 3D crystallographic structure of DNA (PDNB ID: 1W0T) was extracted from the protein data bank^58^ and imported to the MOE window and protonated 3D for docking simulations using MOPAC7.0. The water molecules in the XRD structure of the DNA were not removed to carry out simulations in a solvent environment. The docking simulation was carried out using the finder site under default parameters with an RMS gradient of 0.01 kcal mol^−1^. The docked conformation with minimum free energy and maximum stability was selected for the final calculation of the binding strength.

#### 3.4.2 Experimental procedure

##### 3.4.2.1 UV-vis absorption spectroscopic studies

Absorption spectroscopic studies were carried out using a PerkinElmer Lambda 35 UV-vis spectrometer. Stock solutions (500 μM) of the compounds were prepared in DMSO, and for further dilutions (100 μM), deionized water was used. Fish sperm DNA (st)DNA was prepared in Tris–HCl buffer solution (0.6 M HCl) and 50 mM NaCl with pH = 7.33, and this buffered DNA was filtered using the syringe filtration method to make it protein-free. The solution of (st)DNA gave a ratio of UV absorbance at 260 and 280 nm, *A*_260_/*A*_280_ of 1.9, indicating that (st)DNA was sufficiently free of proteins.^59^ The concentration of (st)DNA was calculated from its absorption intensity at 260 nm with a molar extinction coefficient of 6600 cm^−1^.^60^ The solution of (st)DNA and drug (compounds) was incubated for 5 min before recording the absorption spectrum. A fixed quantity (3 mL) of the compound (100 μM) was titrated with an increasing quantity (10–80 μL) of DNA. The intrinsic binding constants (*k*_b_) were calculated using the following equation:
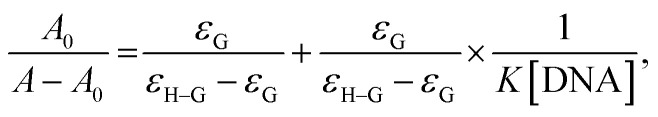
where [DNA] represents the (st)DNA concentration in base molarity, *A*_0_ represents the absorbance of the drug in the absence of DNA, and *A* represents the absorbance of the drug in the presence of DNA. *ε*_G_ and *ε*_H–G_ represent the absorption coefficients of the drug and drug–DNA complex, respectively. The intrinsic binding constant *K*_b_ can be calculated from the intercept to the slope ratio of *A*_0_/*A* − *A*_0_*vs.* 1/[DNA] plots.

##### 3.4.2.2 Thermal stability of DNA

DNA thermal stability as a function of temperature was investigated by absorbance *versus* temperature curve. The change in absorbance with the temperature at 260 nm was monitored from 30 °C to 90 °C at a 1 °C per minute scan rate and fixed [drug]/[DNA] concentration ratio. Accordingly, the DNA melting temperature (*T*_m_) was recorded, and the difference between *T*_m_ values in the absence and presence of compound (Δ*T*_m_) was calculated.

### 3.5 Biochemistry

#### 3.5.1 Antibacterial assay

The synthesized compounds were tested for their antibacterial activity against two Gram-negative, *Escherichia coli* (ATCC 15224), *Enterobacter aerogenes* (ATCC 13048), and two Gram-positive, *Micrococcus luteus* (ATCC 10240), *Staphylococcus aureus* (ATCC 6538), bacterial strains. The disc diffusion method was applied to carry out these biological investigations.^61^ The bacteria were cultured in nutrient broth under controlled temperature conditions (37 °C) for 24 h. From this cultured medium, 1% broth containing about 10^6^ colony-forming units (CFU mL^−1^) of bacterial strain was transferred to the nutrient agar medium at 45 °C and then decanted into the pre-sterilized Petri plates. Upon the solidification of the medium, about 5 μL of the test compound (40 mg mL^−1^ in DMSO) was decanted onto the pre-sterilized paper discs of 4 mm size and positioned on nutrient agar plates. In every plate, 5 μL of the standard antibacterial drug kanamycin (200 μg mL^−1^) served as a positive control, while DMSO served as a negative control. The plates were prepared in triplicate for each bacterial strain and incubated at 37 °C for 24 h. The antibacterial activity was evaluated by measuring the complete inhibition zone diameter (mm) when the concentration of compounds was 200 μg mL^−1^. MIC values were calculated by applying a linear regression method.

#### 3.5.2 Brine shrimp cytotoxic assay

The cytotoxicity of all the compounds (L_1_, L_2_, and complexes 1–8) was assessed by brine shrimp cytotoxic assay.^62^ Eggs of brine shrimp (Artemia) (Ocean Star Inc., USA) were hatched in a rectangular container in artificial seawater that was prepared by a commercial salt mixture in double-distilled water. After 24 h, phototropic nauplii (brine shrimp larvae) were transferred to a glass vial using a Pasteur pipette, and 25 μL of each stock solution (200 μg mL^−1^, 66.6 μg mL^−1^, 22.2 μg mL^−1^, 7.4 μg mL^−1^, and μg mL^−1^) of the test compound was poured into the vial. The volume of the test compound was increased to 5 mL with artificial seawater. DMSO and Doxorubicin (200 μg mL^−1^) were used as negative and positive controls, respectively; three replicates were prepared for each concentration. The vials were maintained under illumination at room temperature. After 24 h, survivors were counted using a 3× magnifying glass, and LD_50_ (Lethal dose that killed 50% of shrimps) was determined using Finny (1971) software.

#### 3.5.3 Cell viability assay

Cytotoxic activity was measured *in vitro* for the newly synthesized compounds the sulfo Rhodamine-B stain (SRB) assay and the method of Skehan at the Institute of Biomedical and Genetic Engineering Islamabad, Pakistan. The test compounds were dissolved in DMSO and diluted with saline to the appropriate volumes. Various concentrations of the compound under tests (1.0, 2.0, 3.0, 4.0 and 5.0 ppm) were added to the cell monolayer. Cisplatin and doxorubicin, standard and well-known anticancer drugs, were used as standard drugs to evaluate the relative cytotoxic efficiency of the tested compounds at the same concentrations. The relation between the surviving fraction and drug concentration is plotted to obtain the survival graphs. The results of cytotoxic activity were expressed as percent cell viability.

## 4. Conclusion

In search of DNA targeting antimicrobial agents, two novel benzimidazoles “O” and “N” donor Schiff base ligands and their complexes with various transition metals were successfully synthesized and characterized by various spectroscopic techniques. DNA binding affinity of compounds was measured by performing UV/Vis absorption titrations and thermal denaturation experiments. Based on the obtained results, it can be concluded that the ligands, and most of their complexes, can bind with DNA by intercalation and groove binding mode as evidenced by the binding constant value, ranging from 10^3^ to 10^5^ M^−1^. Among all the complexes, compound 2 was stronger (*K*_b_, 3.27 × 10^5^ M^−1^) and compound 5 was weaker (*K*_b_, 6.4 × 10^3^ M^−1^) DNA binder. The *in vitro* antibacterial studies indicated that various compounds have the potential to inhibit the growth of bacteria, and compound 2 showed broad-spectrum antibacterial potency against *M. luteus*, *S.aureus*, *E. coli* and *E. aerogenes* with inhibition zone values of 8.5, 10.3, 16.8 and 14.5 mm. The preliminary cytotoxic effect of these compounds was studied on shrimp larvae, and most of the compounds were declared to be non-toxic towards normal cells. Only two compounds, L_1_ and 7, showed slight toxicity with LD_50_ values of 127 and 125 μg mL^−1^, respectively. The compounds showed a marked cytotoxic effect against human breast cancer cells. This leads to the conclusion that the synthesized compounds possessed significant anticancer activity comparable to the activity of commonly used anticancer drugs, cisplatin and doxorubicin. According to the obtained results, the design and synthesis of benzimidazole Schiff base-transition metal complexes were of immense importance to produce novel antimicrobial drug libraries with fewer or no side effects and to expunge the concept of multidrug resistance. Through this study, we want to explain that heterocyclic Schiff base ligands and complexes have all the inherent properties that are required to act as DNA targeting antimicrobial drugs.

## Conflicts of interest

Authors have no conflict of interest for this research work.

## Supplementary Material

RA-013-D3RA00982C-s001

RA-013-D3RA00982C-s002

## References

[cit1] Barone G., Terenzi A., Lauria A., Almerico A. M., Leal J. M., Busto N., García B. (2013). DNA-binding of nickel(II), copper(II) and zinc(II) complexes: Structure-affinity relationships. Coord. Chem. Rev..

[cit2] Rosenberg B., Van Camp L., Krigas T. (1965). Inhibition of cell division in Escherichia coli by electrolysis products from a platinum electrode. Nature.

[cit3] Rosenberg B., Van Camp L., Grimley E. B., Thompson A. J. (1967). The Inhibition of Growth or Cell Division in Escherichia by Different Ionic Species of Platinum(IV) Complexes. J. Biol. Chem..

[cit4] Wee H. A., Dyson P. J. (2006). Classical and non-classical ruthenium-based anticancer drugs: Towards targeted chemotherapy. Eur. J. Inorg. Chem..

[cit5] Aird R. E., Cummings J., Ritchie A. A., Muir M., Morris R. E., Chen H., Sadler P. J., Jodrell D. I. (2002). In vitro and in vivo activity and cross resistance profiles of novel ruthenium(II) organometallic arene complexes in human ovarian cancer. Br. J. Cancer.

[cit6] Park S. Y., Yoon S. J., itiroh Hakomori S., Kim J. M., Kim J. Y., Bernert B., Ullman T., Itzkowitz S. H., Kim J. H. (2010). Dimeric Le(a) (Le(a)-on-Le(a)) status of beta-haptoglobin in sera of colon cancer, chronic inflammatory disease and normal subjects. Int. J. Oncol..

[cit7] Romero-Canelón I., Sadler P. J. (2013). Next-generation metal anticancer complexes: Multitargeting via redox modulation. Inorg. Chem..

[cit8] Liao L. S., Chen Y., Mo Z. Y., Hou C., Su G. F., Liang H., Chen Z. F. (2021). Ni(II), Cu(II) and Zn(II) complexes with the 1-trifluoroethoxyl-2,9,10-trimethoxy-7-oxoaporphine ligand simultaneously target microtubules and mitochondria for cancer therapy. Inorg. Chem. Front..

[cit9] Hernández-Romero D., Rosete-Luna S., López-Monteon A., Chávez-Piña A., Pérez-Hernández N., Marroquín-Flores J., Cruz-Navarro A., Pesado-Gómez G., Morales-Morales D., Colorado-Peralta R. (2021). First-row transition metal compounds containing benzimidazole ligands: An overview of their anticancer and antitumor activity. Coord. Chem. Rev..

[cit10] Farrell T. J. M. N. , in Comprehensive Coordination Chemistry II, ed. J. A. McCleverty, Pergamum, Oxford, 2003, p. 809

[cit11] Holder A. A. (2013). Inorganic pharmaceuticals. Annu. Rep. Prog. Chem., Sect. A: Inorg. Chem..

[cit12] Ronconi L., Sadler P. J. (2007). Using coordination chemistry to design new medicines. Coord. Chem. Rev..

[cit13] Schwietert C. W., McCue J. P. (1999). Coordination compounds in medicinal chemistry. Coord. Chem. Rev..

[cit14] Hussain A., AlAjmi M. F., Md T. R., Khan A. A., Shaikh P. A., Khan R. A. (2018). Evaluation of transition metal complexes of benzimidazole-derived scaffold as promising anticancer chemotherapeutics. Molecules.

[cit15] Alterhoni E., Tavman A., Hacioglu M., Şahin O., Seher Birteksöz Tan A. (2021). Synthesis, structural characterization and antimicrobial activity of Schiff bases and benzimidazole derivatives and their complexes with CoCl2, PdCl2, CuCl2 and ZnCl2. J. Mol. Struct..

[cit16] Aragón-Muriel A., Liscano Y., Upegui Y., Robledo S. M., Teresa Ramírez-Apan M., Morales-Morales D., Oñate-Garzón J., Polo-Cerón D., Sinicropi S. (2021). In Vitro Evaluation of the Potential Pharmacological Activity and Molecular Targets of New Benzimidazole-Based Schiff Base Metal Complexes. Antibiotics.

[cit17] Goudgaon N. M. (2015). Synthesis , characterization and biological evaluation of novel C-2 substituted benzimidazole heterocycles. J. Pharm. Res..

[cit18] Fei F., Zhou Z. (2013). New substituted benzimidazole derivatives: a patent review (2010 – 2012). Expert Opin. Ther. Pat..

[cit19] Raman N., Selvan A. (2012). Investigation of DNA binding mechanism, photoinduced cleavage activity, electrochemical properties and biological functions of mixed ligand copper(II) complexes with benzimidazole derivatives: Synthesis and spectral characterization. J. Enzyme Inhib. Med. Chem..

[cit20] Nykjaer A., Fyfe J. C., Kozyraki R., Leheste J., Jacobsen C., Nielsen M. S., Verroust P. J., Aminoff M., De A., Moestrup S. K., Ray R., Gliemann J., Willnow T. E., Erik I., Verroust P. J., Aminoff M., De Chapelle A., Moestrup S. K., Raytt R. (2016). Induced-fit recognition of DNA by organometallic complexes with dynamic stereogenic centers. Proc. Natl. Acad. Sci..

[cit21] Barone G., Gambino N., Ruggirello A., Silvestri A., Terenzi A., Liveri V. T. (2009). Spectroscopic study of the interaction of NiII-5-triethyl ammonium methyl salicylidene ortho-phenylendiiminate with native DNA. J. Inorg. Biochem..

[cit22] Silvestri A., Barone G., Ruisi G., Anselmo D., Riela S., Liveri V. T. (2007). The interaction of native DNA with Zn(II) and Cu(II) complexes of 5-triethyl ammonium methyl salicylidene ortho-phenylendiimine. J. Inorg. Biochem..

[cit23] Mansour A. M., Shehab O. R. (2018). Lysozyme and DNA binding affinity of Pd(II) and Pt(II) complexes bearing charged N,N pyridylbenzimidazole bidentate ligands. Dalton Trans..

[cit24] Ibrahim M. A., El-Mahdy K. M. (2009). Synthesis and antimicrobial activity of some new heterocyclic schiff bases derived from 2-Amino-3-formylchromone. Phosphorus, Sulfur Silicon Relat. Elem..

[cit25] Reddy K. R., Reddy K. M., Mahendra K. N. (2006). Synthesis, characterization, antibacterial and anthelmentic activities of copper (II) complexes with benzofuran Schiff bases. Indian J. Chem..

[cit26] Chohan Z. H., Shad H. A., Supuran C. T. (2012). Synthesis, characterization and biological studies of sulfonamide Schiff's bases and some of their metal derivatives. J. Enzyme Inhib. Med. Chem..

[cit27] Sathiyaraj S., Ayyannan G., Jayabalakrishnan C. (2014). Synthesis, spectral, DNA binding and cleavage properties of ruthenium(II) Schiff base complexes containing PPh3/AsPh3 as co-ligands. J. Serb. Chem. Soc..

[cit28] Ma L., Li L., Zhu G. (2022). Platinum-containing heterometallic complexes in cancer therapy: advances and perspectives. Inorg. Chem. Front..

[cit29] Sadtler S. W. T. , Handbook of Proton NMR Spectra, 1978

[cit30] Pasto D. J. , Organic structure determination, 1969

[cit31] Cavasotto C. N. (2006). Ligand docking and virtual screening in structure-based drug discovery. AIP Conf. Proc..

[cit32] Caruso F., Rossi M., Benson A., Opazo C., Freedman D., Monti E., Gariboldi M. B., Shaulky J., Marchetti F., Pettinari R., Pettinari C. (2012). Ruthenium-arene complexes of curcumin: X-ray and density functional theory structure, synthesis, and spectroscopic characterization, in vitro antitumor activity, and DNA docking studies of (p-cymene)Ru(curcuminato)chloro. J. Med. Chem..

[cit33] Ferreira L. G. , Santos R. N., Oliva G. and Andricopulo A. D., Dms43_Vitae[1].Pdf, 2015

[cit34] Kosiha A., Parthiban C., Ciattini S., Chelazzi L., Elango K. P. (2017). Metal complexes of naphthoquinone based ligand: synthesis, characterization, protein binding, DNA binding/cleavage and cytotoxicity studies. J. Biomol. Struct. Dyn..

[cit35] Parveen M., Malla A. M., Yaseen Z., Ali A., Alam M. (2014). Synthesis, characterization, DNA-binding studies and acetylcholinesterase inhibition activity of new 3-formyl chromone derivatives. J. Photochem. Photobiol., B.

[cit36] Fukuda R., Takenaka S., Takagi M. (1990). Metal ion assisted DNA-intercalation of crown ether-linked acridine derivatives. J. Chem. Soc., Chem. Commun..

[cit37] Sakthi M., Ramu A. (2017). Synthesis, structure, DNA/BSA binding and antibacterial studies of NNO tridentate Schiff base metal complexes. J. Mol. Struct..

[cit38] Basu A., Suresh Kumar G. (2015). Thermodynamic characterization of proflavine-DNA binding through microcalorimetric studies. J. Chem. Thermodyn..

[cit39] Wells A. F. , Structural Inorganic Chemistry, Clarendon Press, Oxford, 5th edn, 1984

[cit40] Xiao S., Lin W., Wang C., Yang M. (2001). Synthesis and biological evaluation of DNA targeting flexible side-chain substituted β-carboline derivatives. Bioorg. Med. Chem. Lett..

[cit41] Cory M., Mckee D. D., Kagan J., Henry D. W., Milled J. A. (1985). Bifunctional Intercalators. Comparison of Polymethylene. J. Am. Chem. Soc..

[cit42] V Kumar C., Asuncion E. H. (1993). DNA Binding Studies and Site Selective Fluorescence Sensitization of an Anthryl Probe. J. Am. Chem. Soc..

[cit43] Selvakumar B., Rajendiran V., Uma Maheswari P., Stoeckli-Evans H., Palaniandavar M. (2006). Structures, spectra, and DNA-binding properties of mixed ligand copper(II) complexes of iminodiacetic acid: The novel role of diimine co-ligands on DNA conformation and hydrolytic and oxidative double strand DNA cleavage. J. Inorg. Biochem..

[cit44] An Y., Liu S. D., Deng S. Y., Ji L. N., Mao Z. W. (2006). Cleavage of double-strand DNA by linear and triangular trinuclear copper complexes. J. Inorg. Biochem..

[cit45] Neyhart G. A., Grover N., Smith S. R., Kalsbeck W. A., Fairley T. A., Cory M., Thorp H. H. (1993). Binding and kinetics studies of oxidation of DNA by Oxoruthenium(IV). J. Am. Chem. Soc..

[cit46] Osowole A. A., Kempe R., Schobert R. (2012). Synthesis , Spectral , Thermal , In-vitro Antibacterial and Anticancer Activities of some Metal (II) Complexes of 3- (1- (4-methoxy-6-). Int. Res. J. Pure Appl. Chem..

[cit47] Ceyhan G., Çelik C., Uruş S., Demirtaş I., Elmastaş M., Tümer M. (2011). Antioxidant, electrochemical, thermal, antimicrobial and alkane oxidation properties of tridentate Schiff base ligands and their metal complexes. Spectrochim. Acta, Part A.

[cit48] Bertini H. S. I. and Sigel A., Handbook on Metalloproteins, Marcel Dekker, New York, 2001

[cit49] Gajbhiye S. N., Hirota R. (1990). Toxicity of Heavy Metals To Brine Shrimp Artemia. J. Indian Fish. Assoc..

[cit50] Lacroix J., Patenaude A., Rousseau J. L. C. (2008). N -Phenyl-N0-(2-chloroethyl)ureas (CEUs) as potential antineoplastic agents. Part 3: Role of carbonyl groups in the covalent binding to the colchicine-binding site. Bioorg. Med. Chem..

[cit51] Thati B., Noble A., Creaven B. S., Walsh M., Mccann M., Kavanagh K., Devereux M. (2007). In vitro anti-tumour and cyto-selective effects of coumarin-3-carboxylic acid and three of its hydroxylated derivatives , along with their silver-based complexes, using human epithelial carcinoma cell lines. Cancer Lett..

[cit52] Creaven B. S., Duff B., Egan D. A., Kavanagh K., Rosair G., Reddy V., Walsh M. (2010). Inorganica Chimica Acta Anticancer and antifungal activity of copper (II) complexes of quinolin-2 (1H) -one-derived Schiff bases. Inorg. Chim. Acta.

[cit53] Taylor P. (2014). Synthesis, Characterization , and Antiproliferative Activity of Cu , V (IV) O , Co , Mn , and Ni Complexes with 3- ( 2- ( 4- Methoxyphenylcarbamothioyl)Hydrazinyl)-3-OXO-N-(Thiazol-2-yl). Phosphorus, Sulfur Silicon Relat. Elem..

[cit54] Mahmood K., Hashmi W., Ismail H., Mirza B. (2018). Synthesis , DNA binding and antibacterial activity of metal (II) complexes of a benzimidazole Schiff base. Polyhedron.

[cit55] Petering D. H., Byrnes R. W., Antholine W. E. (1992). Oxidation-reduction reactions in ehrlich cells treated with copper-neocuproine. Free Radical Biol. Med..

[cit56] Sheldrick G. M. (2008). A short history of SHELX. Acta Crystallogr., Sect. A: Found. Crystallogr..

[cit57] Spek A. (2009). Structure validation in chemical crystallography. Acta Crystallogr., Sect. D: Biol. Crystallogr..

[cit58] https://www.rcsb.org/structure/1w0t

[cit59] El-Deen I. M., Shoair A. F., El-Bindary M. A. (2018). Synthesis, structural characterization, molecular docking and DNA binding studies of copper complexes. J. Mol. Liq..

[cit60] Reichmann M. E., Rice C. A., Thomas C. A., Doty P. (1954). A further examination of the molecular weight and size of deoxypentose nucleic acid. J. Am. Chem. Soc..

[cit61] Zaheer M., Shah A., Akhter Z., Qureshi R., Mirza B., Tauseef M., Bolte M. (2011). Synthesis, characterization, electrochemistry and evaluation of biological activities of some ferrocenyl Schiff bases. Appl. Organomet. Chem..

[cit62] Ul-Haq I., Ullah N., Bibi G., Kanwal S. (2012). Antioxidant and cytotoxic activities and phytochemical analysis of Euphorbia wallichii root extract and its fractions. Pharm. Res..

