# Supplementary material for: Synthesis, DNA binding and biological evaluation of benzimidazole Schiff base ligands and their metal(ii) complexes
Source: RSC Adv. 2023 Apr 17;13(18):11982–99. doi: 10.1039/d3ra00982c (PMC10107730; doi:10.1039/d3ra00982c)
Supplement: RA-013-D3RA00982C-s001 [file RA-013-D3RA00982C-s001.pdf]

Electronic Supplementary Material (ESI) for RSC Advances.
This journal is © The Royal Society of Chemistry 2023

Supplementary Data

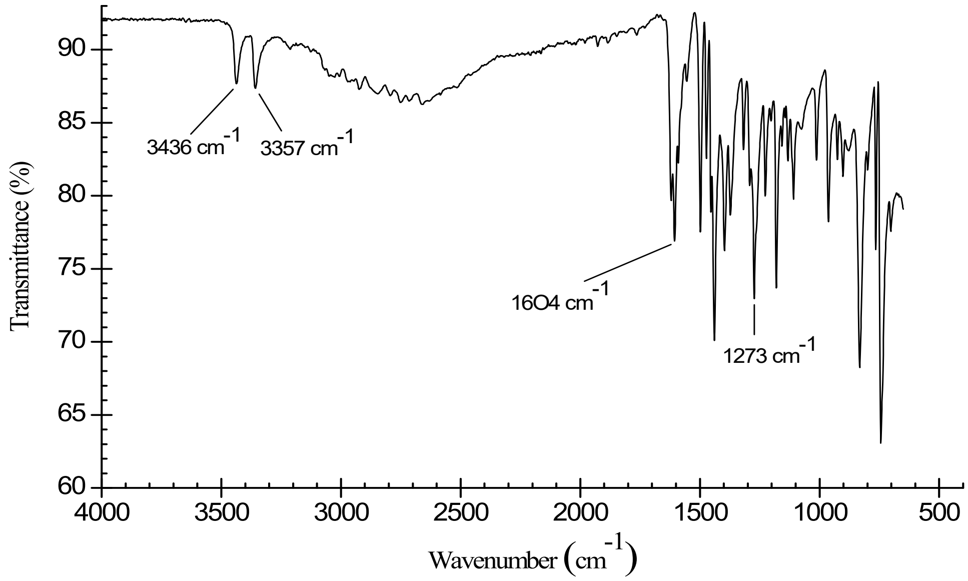

Figure S1. Infrared spectrum of A-**1**

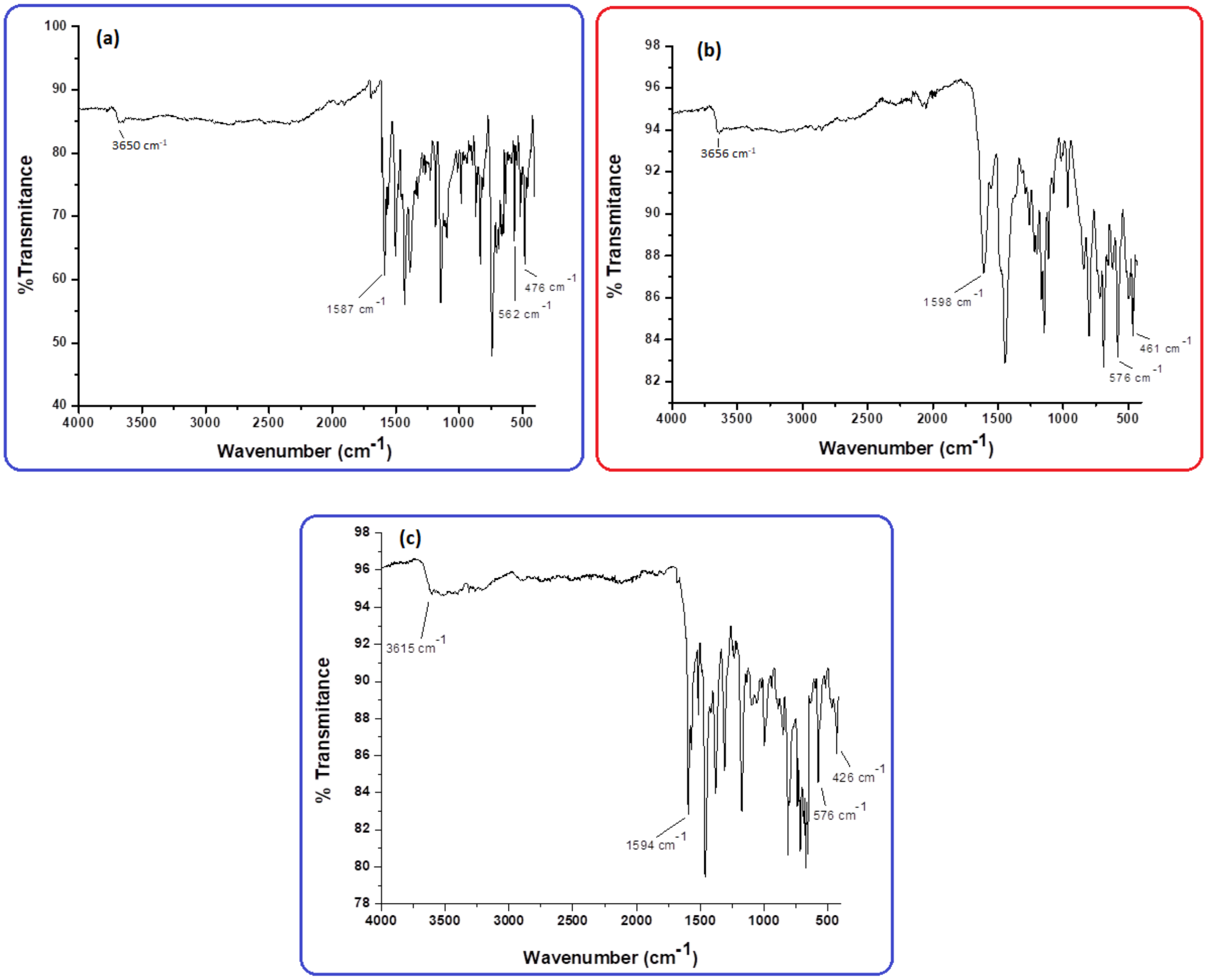

Figure S2. Infrared spectra of Complexes 2 (a), 3 (b) and 4 (c)

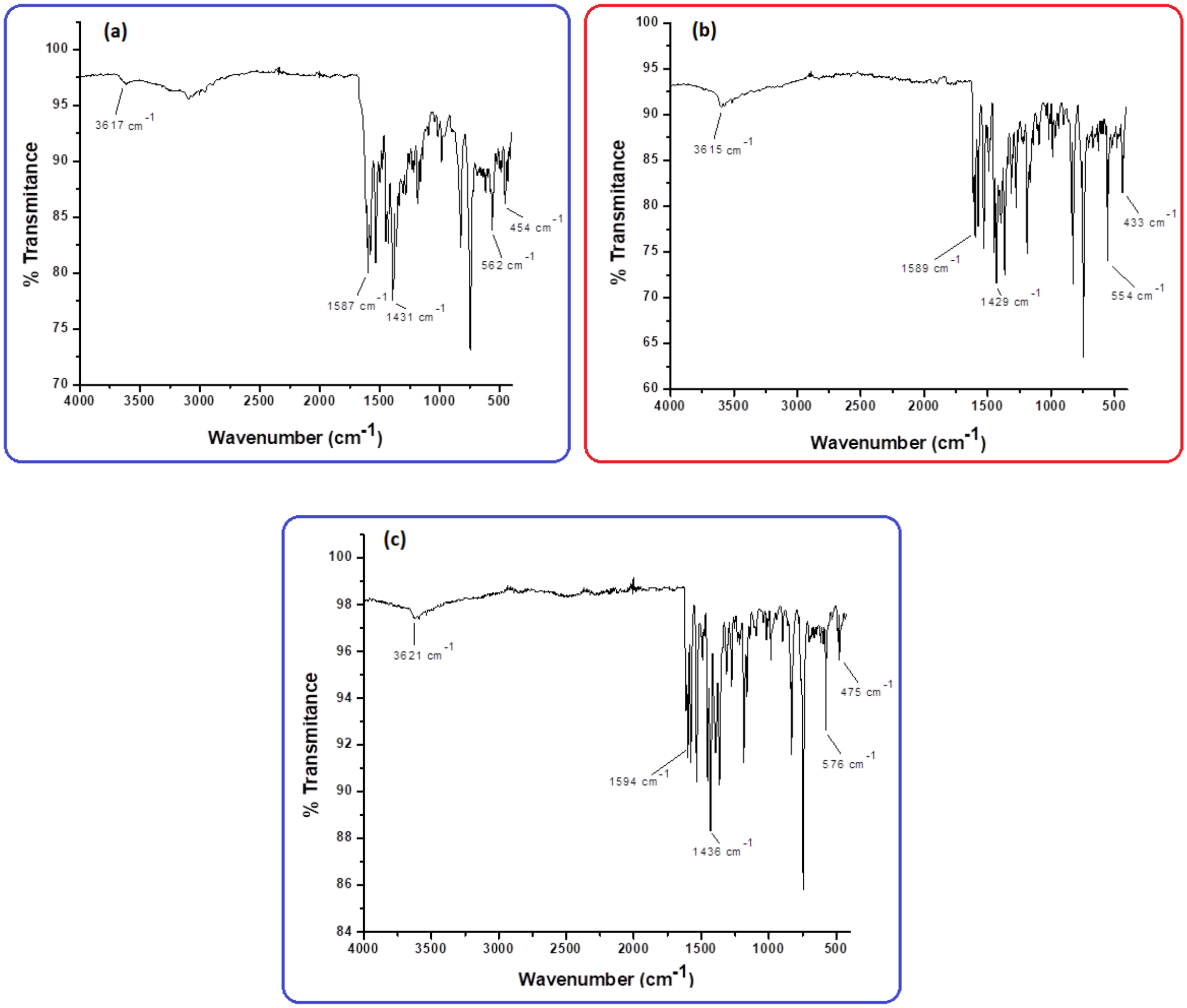

Figure S3. Infrared spectra of Complexes 6 (a), 7 (b) and 8 (c)

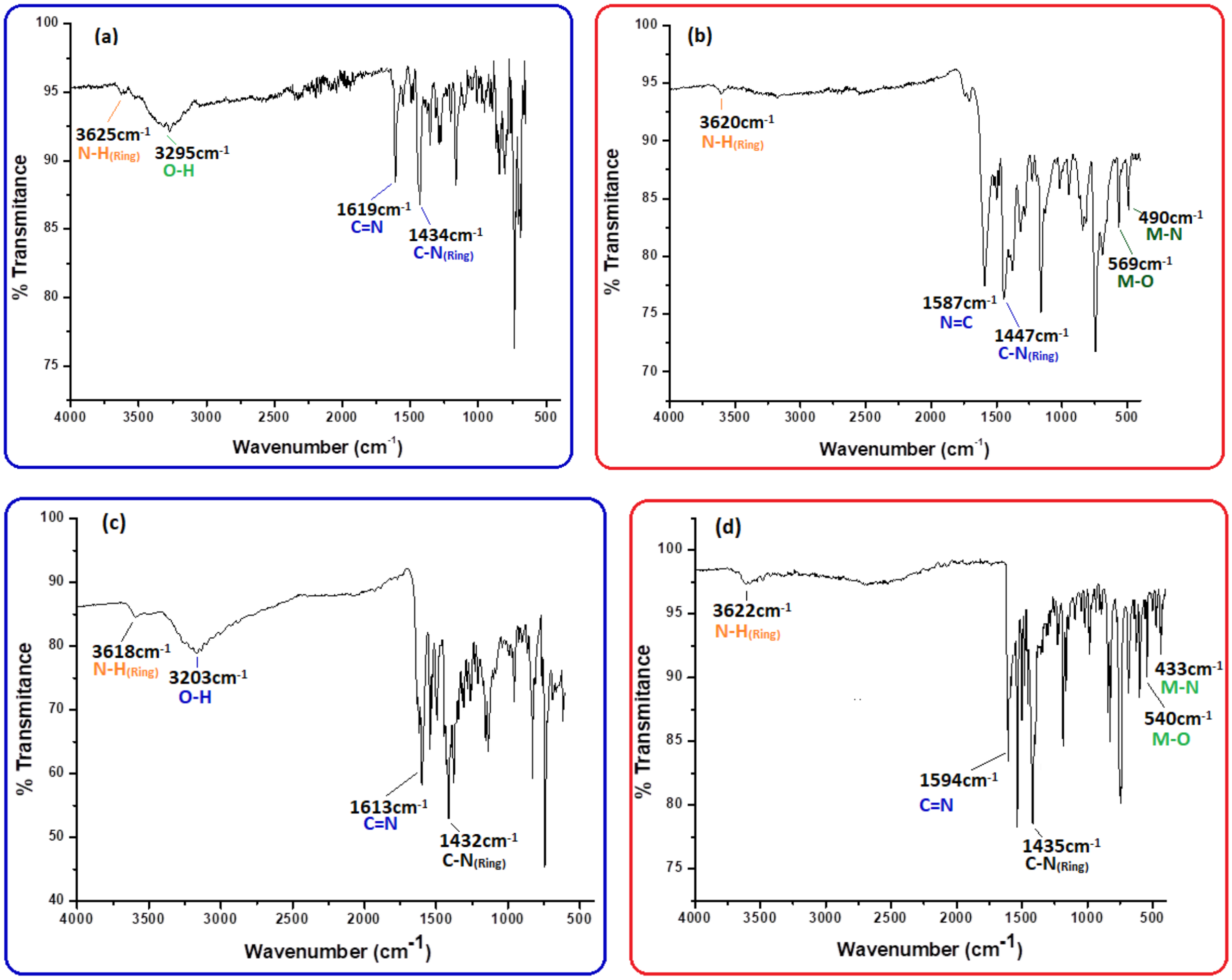

Fig. S4. Representative FT-IR spectra of ligands **L$_1$** (a), **L$_2$** (c) and Cu(II) complexes (**1**) (b), **5** (d).

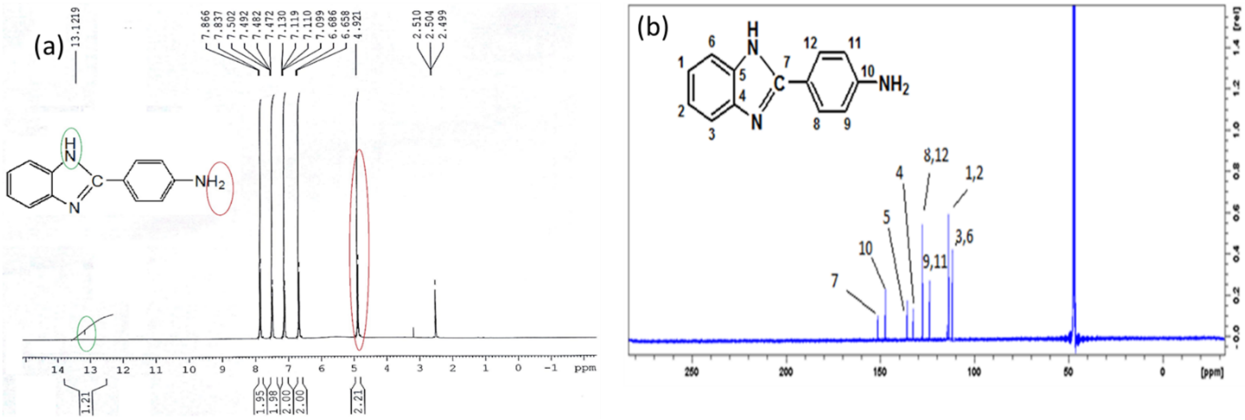

Figure S5. ^{1}H NMR (a) and ^{13}C NMR (b) spectra of **A-1** in DMSO-d^6

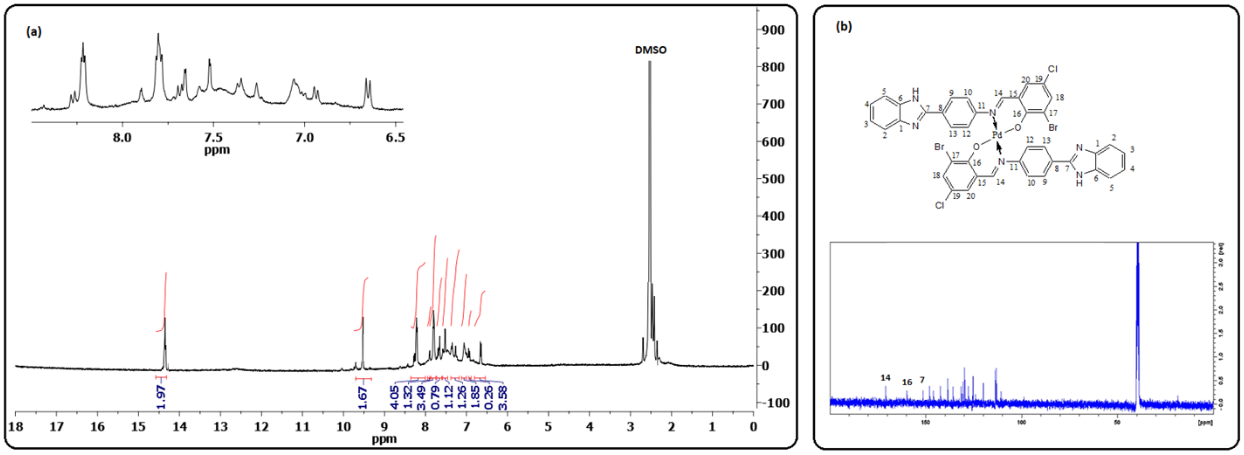

Figure S6. ^{1}H NMR (a) and ^{13}C NMR (b) spectra of **Ni(II)** complex **2** in DMSO-d^{6}

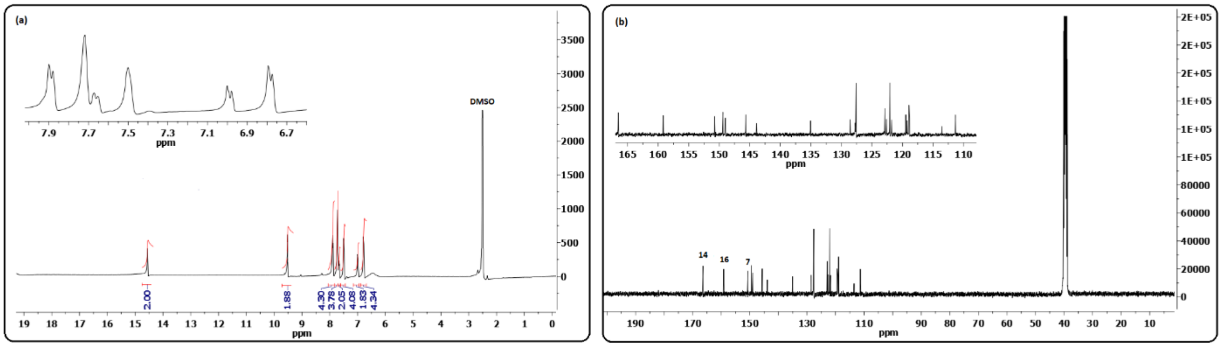

Figure S7. ^{1}H NMR (a) and ^{13}C NMR (b) spectra of **Pd(II)** complex **3** in DMSO-d^{6}

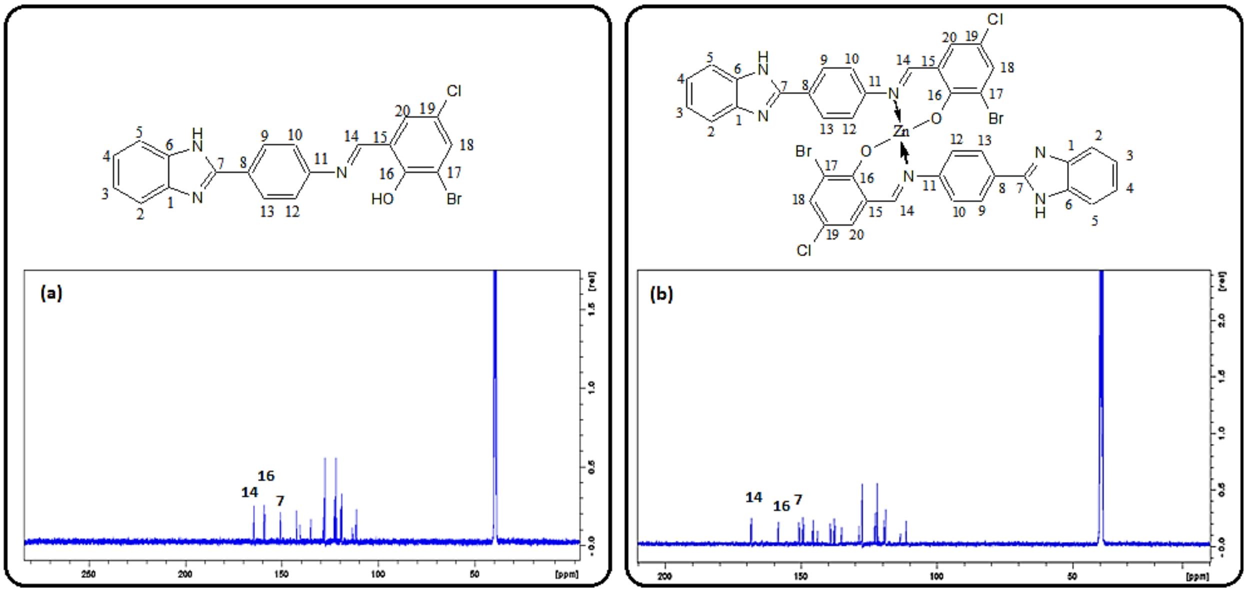

Figure S8. ^{13}C NMR spectrum of ligand L_1 (a) and Zn(II) complex **4** (b) in DMSO-d^{6}

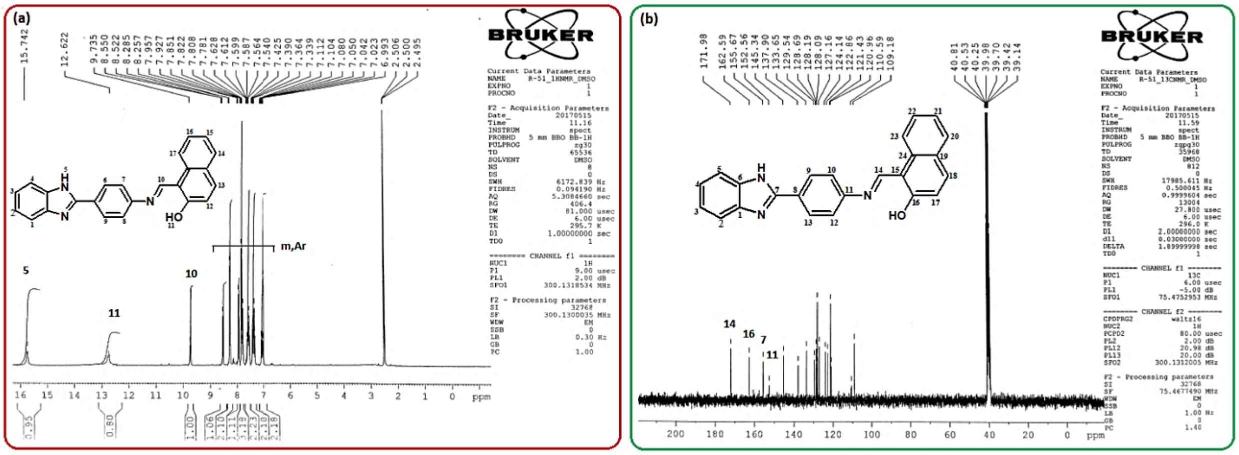

Figure S9. ^{1}H NMR (a) and ^{13}C NMR (b) spectra of ligand L_2 in DMSO-d^6

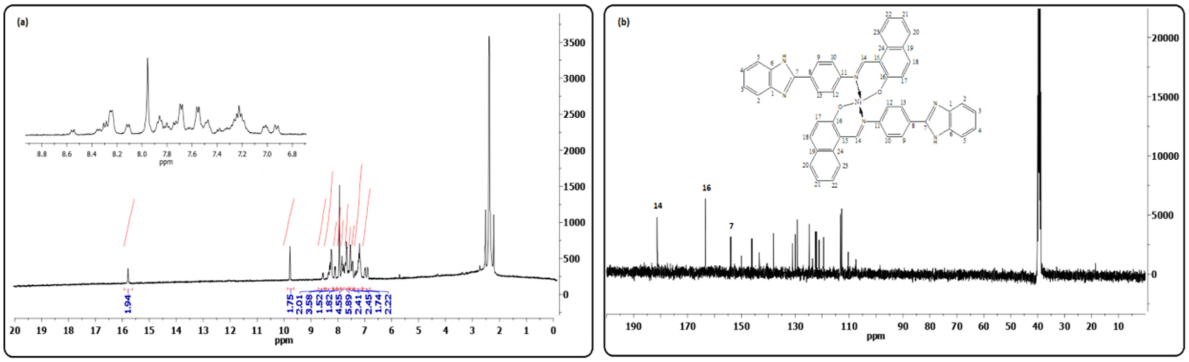

Figure S10. ^{1}H NMR (a) and ^{13}C NMR (b) spectra of **Zn(II)** complex **6** in DMSO-d^6

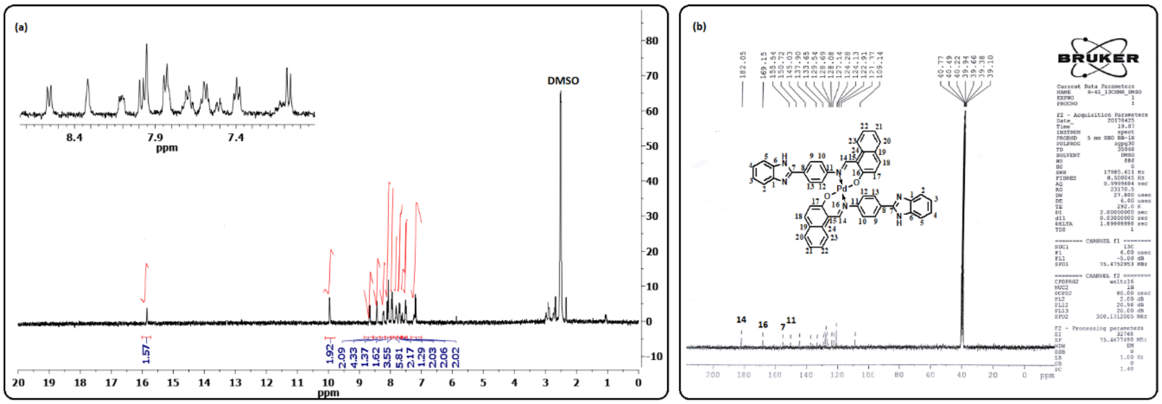

Figure S11. ^{1}H NMR (a) and ^{13}C NMR (b) spectra of **Pd(II)** complex **7** in DMSO-d^6

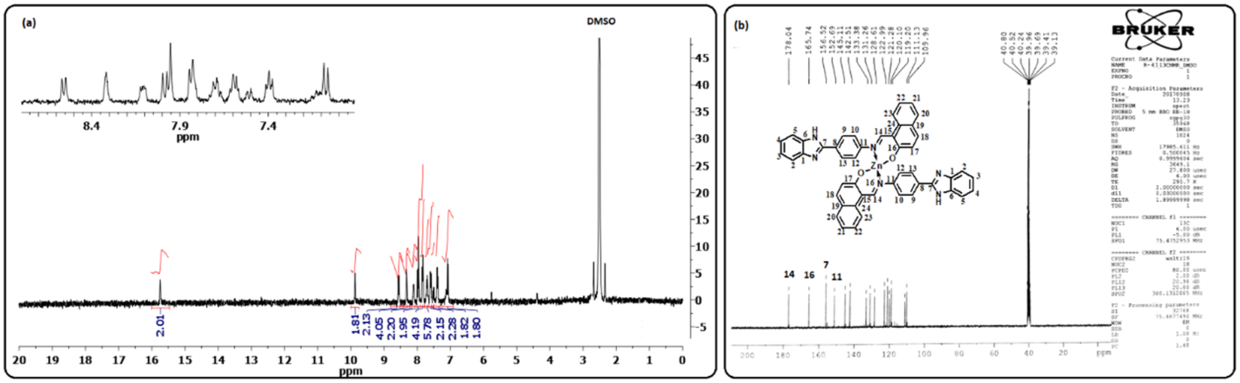

Figure S12. ^{1}H NMR (a) and ^{13}C NMR (b) spectra of **Zn(II)** complex **8** in DMSO-d^{6}

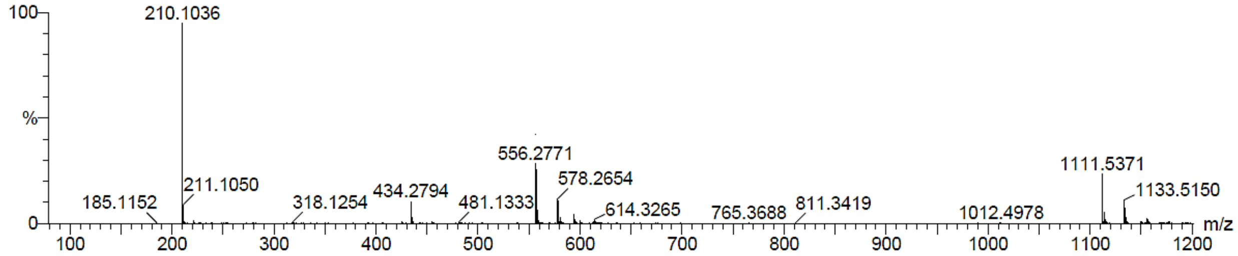

Minimum:				0.5		
Maximum:		5.0	10.0	100.0		
Mass	Calc. Mass	mDa	PPM	DBE	i-FIT	Formula
210.1036	210.1031	0.5	2.4	9.5	201.6	C13 H12 N3

Figure S13. High resolution mass spectrum of **A-1**

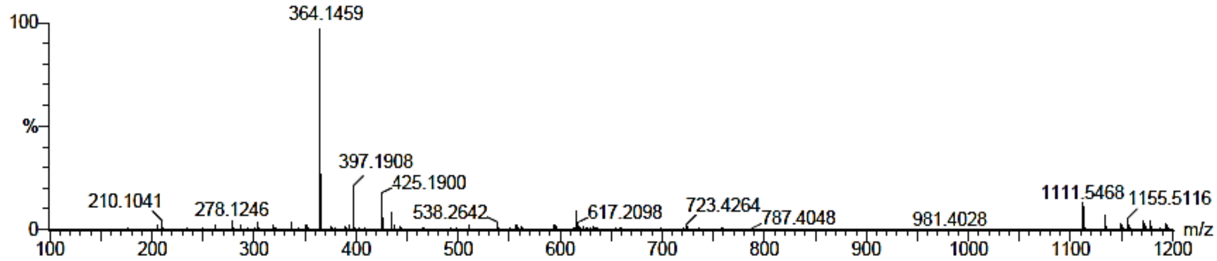

Minimum:				0.5		
Maximum:		5.0	10.0	200.0		
Mass	Calc. Mass	mDa	PPM	DBE	i-FIT	Formula
364.1459	364.1450	0.9	2.5	17.5	3.5	C24 H18 N3 O

Figure S14. High Resolution Mass spectrum of L_2

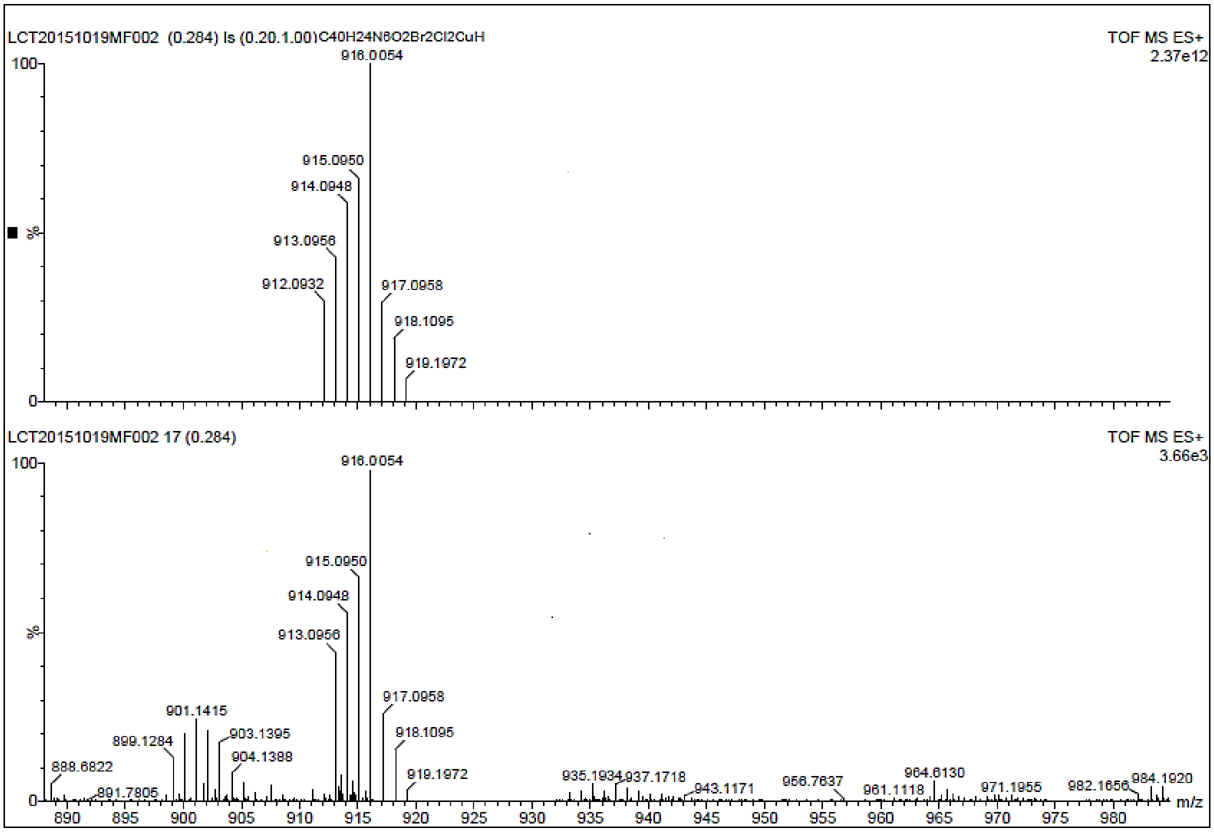

Figure S15. High Resolution Mass spectrum of complex 1

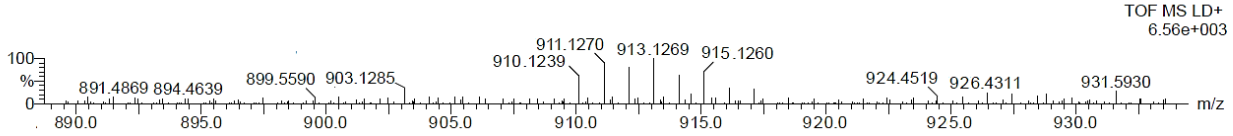

Minimum:				-1.5			
Maximum:		5.0	200.0	200.0			
Mass	Calc. Mass	mDa	PPM	DBE	i-FIT	i-FIT (Norm)	Formula
910.1239	910.1233	3.7	4.3	29.5	181.7	0.0	C40 H24 N6 O2 Cl2 Br2 Ni

Figure S16. High Resolution Mass spectrum of complex 2

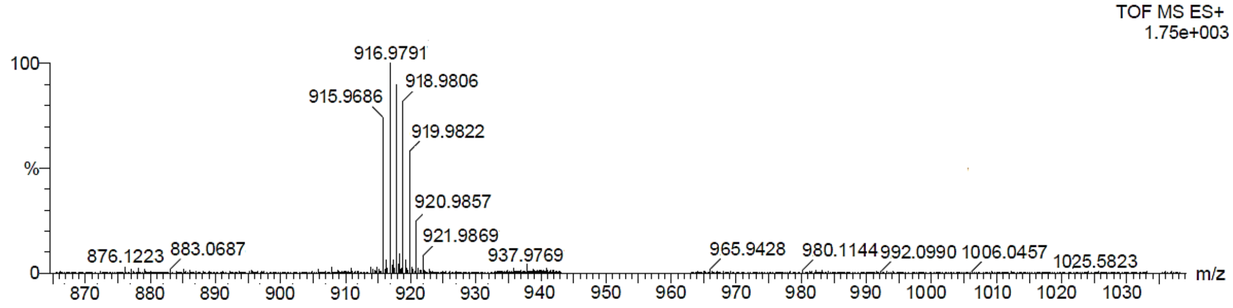

Minimum:				0.5		
Maximum:		5.0	10.0	100.0		
Mass	Calc. Mass	mDa	PPM	DBE	i-FIT	Formula
916.9791	916.9764	-1.5	-2.0	29.5	864.8	C40 H24 N6 O2 Cl2 Br2 Zn

Figure S17. High Resolution Mass spectrum of complex **4**

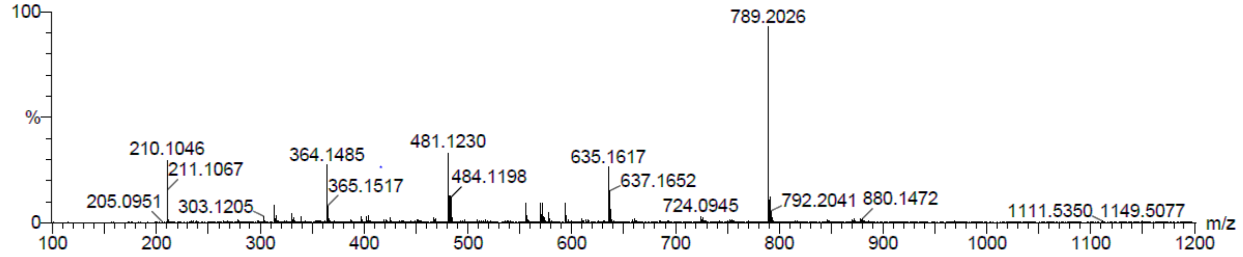

Minimum:				0.5		
Maximum:		5.0	10.0	200.0		
Mass	Calc. Mass	mDa	PPM	DBE	i-FIT	Formula
789.2026	789.2039	-1.3	-1.6	35.0	0.4	C48 H34 N6 O2 Cu

Figure S18. High Resolution Mass spectrum of complex **5**

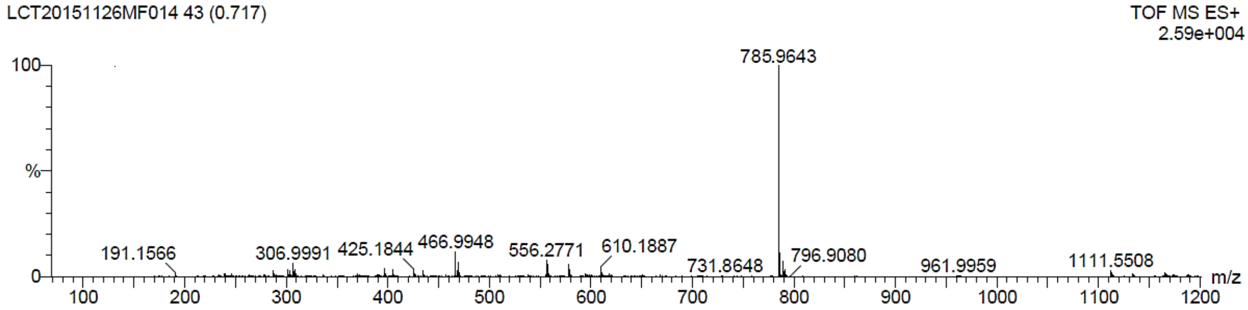

Minimum:				0.5		
Maximum:		5.0	5.0	200.0		
Mass	Calc. Mass	mDa	PPM	DBE	i-FIT	Formula
785.9643	785.9635	-0.1	-0.2	16.0	1.0	C48 H33 N6 O2 Ni

Figure S19. High Resolution Mass spectrum of complex **6**

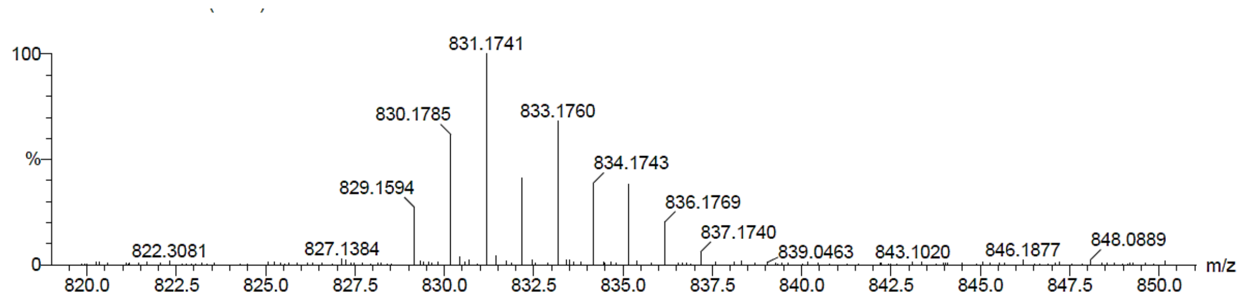

Minimum:				0.5		
Maximum:		5.0	10.0	200.0		
Mass	Calc. Mass	mDa	PPM	DBE	i-FIT	Formula
831.1741	831.1700	4.1	4.9	35.5	1.5	C48 H33 N6 O2 Pd

Figure S20. High Resolution Mass spectrum of complex **7**

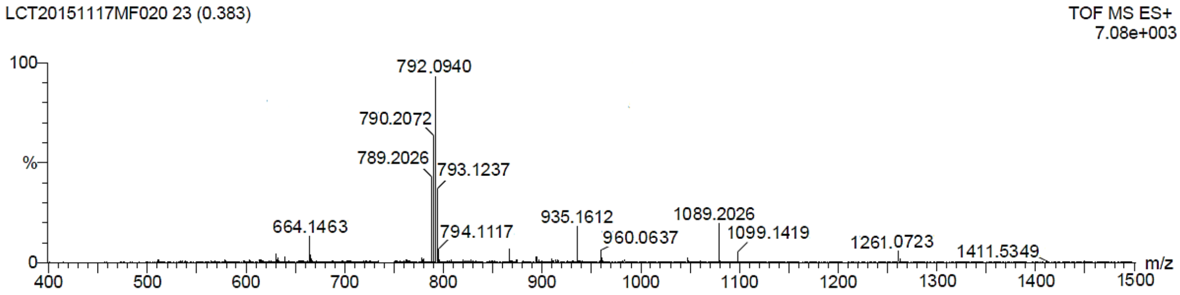

Minimum:				0.5		
Maximum:		5.0	10.0	200.0		
Mass	Calc. Mass	mDa	PPM	DBE	i-FIT	Formula
792.0940	792.0961	-0.5	-1.5	25.0	0.6	C48 H33 N6 O2 Zn

Figure S21. High Resolution Mass spectrum of complex **8**

Table S1: Determination of minimum inhibitory concentration (MIC µg/mL) of test compounds

Compounds	*M. luteus*						*S. aureus*						*E. coli*						*E. aerogenes*					
	a	b	c	d	e	f	a	b	c	d	e	f	a	b	c	d	e	f	a	b	c	d	e	f
L1	-	-	+	+	+	+	+	+	+	+	+	+	-	-	-	+	+	+	-	-	-	+	+	+
L2	+	+	+	+	+	+	+	+	+	+	+	+	-	-	+	+	+	+	-	-	-	+	+	+
1	+	+	+	+	+	+	-	-	+	+	+	+	-	-	-	+	+	+	-	-	+	+	+	+
2	-	-	-	-	+	+	-	-	-	-	+	+	-	-	-	-	-	+	-	-	-	+	+	+
3	-	-	+	+	+	+	+	+	+	+	+	+	+	+	+	+	+	+	+	+	+	+	+	+
4	-	+	+	+	+	+	+	+	+	+	+	+	-	-	-	+	+	+	-	-	-	+	+	+
5	+	+	+	+	+	+	+	+	+	+	+	+	+	+	+	+	+	+	+	+	+	+	+	+
6	-	-	-	+	+	+	-	-	+	+	+	+	-	-	-	-	+	+	-	-	+	+	+	+
7	-	-	+	+	+	+	-	-	+	+	+	+	+	+	+	+	+	+	+	+	+	+	+	+
8	-	-	+	+	+	+	+	+	+	+	+	+	-	-	-	+	+	+	+	+	+	+	+	+

a=200 µg/mL, b=100 µg/mL, c=50 µg/mL, d=25 µg/mL, e=12.5 µg/mL, f=6.25 µg/mL

Minus (-) indicate the absence of growth, Plus (+) indicate the presence of growth

Table S2: Results of DPPH free radical scavenging assay

Compounds	Percentage Scavenging					$IC50 µgmL^{-1}$
	200 $µgmL^{-1}$	66.6 $µgmL^{-1}$	22.2 $µgmL^{-1}$	7.4 $µgmL^{-1}$	2.46$µgmL^{-1}$	
L1	52.88	50.23	48.47	45.56	14.38	60.25
L2	52.67	50.94	47.35	42.88	32.39	61.45
1	65.57	53.58	49.44	21.66	11.15	26.47
2	62.39	57.17	51.63	41.01	20.3	19.25
3	78.52	62.11	53.64	23.97	19.71	17.34
4	51.11	50.74	45.2	31.41	12	60.15
5	68.42	59.39	45.32	31.66	28.15	51.21
6	85	67.17	65.63	51.01	40.3	7.1
7	73.8	62.6	62.44	52.34	49.71	3.32
8	50.05	49.32	46.56	38.17	12.81	88.98
Ascorbic acid	82.43	66.59	61.74	55.41	40.31	4.23

Table S3: Results of brine shrimp cytotoxicity assay

Comp.	Percentage mortality after 24 hours			LD_{50} µg/ml
	200µg/ml	66.6µg/ml	22.2µg/ml	
L_1	70	20	20	127.61
L_2	30	10	10	>200
1	40	30	10	>200
2	30	20	10	>200
3	20	10	10	>200
4	-	-	-	-
5	-	-	-	-
6	30	20	10	>200
7	60	40	10	125
8	-	-	-	-
Doxorubicin	95	90	70	3.22
